# Structure Revision of Isocereulide A, an Isoform of the Food Poisoning Emetic *Bacillus cereus* Toxin Cereulide

**DOI:** 10.3390/molecules26051360

**Published:** 2021-03-04

**Authors:** Veronika Walser, Markus Kranzler, Monika Ehling-Schulz, Timo D. Stark, Thomas F. Hofmann

**Affiliations:** 1 Food Chemistry and Molecular Sensory Science, Technical University of Munich, Lise-Meitner-Str. 34, 85354 Freising, Germany; veronika.walser@tum.de (V.W.); thomas.hofmann@tum.de (T.F.H.); 2 Institute of Microbiology, Department of Pathobiology, University of Veterinary Medicine Vienna, Veterinärplatz 1, 1210 Vienna, Austria; Markus.Kranzler@vetmeduni.ac.at (M.K.); Monika.Ehling-Schulz@vetmeduni.ac.at (M.E.-S.)

**Keywords:** *B. cereus*, isocereulide(s), structure elucidation, UPLC-MS, NMR spectroscopy, MS^n^ sequencing

## Abstract

The emetic *Bacillus cereus* toxin cereulide presents an enormous safety hazard in the food industry, inducing emesis and nausea after the consumption of contaminated foods. Additional to cereulide itself, seven structurally related isoforms, namely the isocereulides A–G, have already been elucidated in their chemical structure and could further be identified in *B. cereus* contaminated food samples. The newly performed isolation of isocereulide A allowed, for the first time, 1D- and 2D-NMR spectroscopy of a biosynthetically produced isocereulide, revealing results that contradict previous assumptions of an l-*O*-Leu moiety within its chemical structure. By furthermore applying posthydrolytical dipeptide analysis, amino acid and *α*-hydroxy acid analysis by means of UPLC-ESI-TOF-MS, as well as MS^n^ sequencing, the structure of previously reported isocereulide A could be corrected. Instead of the l-*O*-Leu as assumed to date, one l-*O*-Ile unit could be verified in the cyclic dodecadepsipeptide, revising the structure of isocereulide A to [(d-*O*-Leu-d-Ala-l-*O*-Val-l-Val)_2_(d-*O*-Leu-d-Ala-l-*O*-Ile-l-Val)].

## 1. Introduction

The ubiquitous, endospore-forming, facultative anaerobe bacterium *Bacillus cereus* [1] is commonly known as a food-borne pathogen, causing, among others, emesis in consequence of the production of its emetic toxin cereulide (**1**), which is inert to a wide range of environmental parameters such as temperature, pH values, and enzymes [2,3,4]. The characteristic dodecadepsipeptide structure of cereulide (**1**) is composed of the three times circularly repeating tetradepsipeptide unit l-*O*-Val-l-Val-d-*O*-Leu-d-Ala, leading to a rectangular cylindrical shape [5,6,7,8].

This complex three-dimensional dodecadepsipeptide structure is biosynthetically assembled by nonribosomal peptide synthetases (NRPSs), designated CesNRPS [9]. The CesNRPS genes, which are located on a virulence megaplasmid pXO1 [10,11], are organized as an operon. Apart from the structural cereulide synthetase genes *cesA* and *cesB*, the *ces* gene locus comprises a phosphopantheteintransferase involved in the activation of the NRPS machinery, a type II thioesterase with a proofreading function and an ABC transporter, recently shown not only to be involved in cereulide export but also directly in cereulide biosynthesis [12,13,14]. A peptidyl carrier protein (PCP)-coupled d-*O*-Leu-d-Ala didepsipeptide intermediate is generated by CesA with an in situ reduced d-Leu moiety coupled to d-Ala. Analogously, CesB assembles one in situ reduced l-Val unit with l-Val to form the corresponding l-*O*-Val-l-Val intermediate [15,16]. Cereulide (**1**) biosynthesis then takes place over a generated l-*O*-Val-l-Val-d-*O*-Leu-d-Ala-PCP-coupled intermediate [8] or via the trimerization and macrocyclization of an l-*O*-Val-l-Val-d-*O*-Leu-d-Ala intermediate by the thioesterase (TE)-dependent CesTE. The latter hypothesis aligns the biosynthesis pathway with the structurally similar toxin valinomycin [17,18].

Next to cereulide (**1**), a wide chemodiversity of structural homologs have been reported to be present in all emetic *B. cereus* strains or have been synthetically produced, and moreover, the naturally occurring isocereulides A–G were identified in their structure [19,20,21]. The natural isocereulides, which are produced simultaneously by the CesNRPS, are thought to be formed by one single misincorporation of an *α*-hydroxy acid or an α-amino acid by the subunits A1 and A2 of CesA or CesB [18].

The highly cytotoxic isocereulide A (**2**) was reported to hold one misincorporation in an *α*-hydroxy acid moiety and thus differing from cereulide (**1**) by implementing one l-*O*-Leu instead of one l-*O*-Val unit into its cyclic dodecadepsipeptide structure. Isocereulide A (**2**) was therefore reported as [(d-*O*-Leu-d-Ala-l-*O*-Val-l-Val)_2_(d-*O*-Leu-d-Ala-l-*O*-Leu-l-Val)] [20]. In carrying on the research on cereulide chemodiversity [20] and further investigating the existence of isocereulide variants, a discrepancy in the structure of isocereulide A reported to date was revealed. 1D- and 2D-NMR spectroscopic experiments shed light on a different *α*-hydroxy acid substitution. After full structure characterization of the aforementioned isocereulide by means of UPLC-TOF-MS experiments, ion-trap MS^n^ sequencing, posthydrolytic dipeptide, and enantioselective amino acid analysis, as well as further structure investigation regarding the *α*-hydroxy acid composition, a corrected chemical structure could be elucidated, and thus, the chemical structure of isocereulide A (**2**) was revised.

## 2. Results and Discussion

### 2.1. Mass Spectrometric Characterization and Isolation of Cereulide *(**1**)* and Isocereulide A *(**2**)*

Next to cereulide (**1**; *m/z* 1170.7125, [M+NH_4_]^+^), isocereulide A (**2**; *m/z* 1184.7281, [M+NH_4_]^+^) was isolated to allow structure elucidation via UPLC-ESI-TOF-MS and 1D- and 2D-NMR spectroscopy. Cereulide (**1**) and isocereulide A (**2**), isolated from cell material originating from a culture of the *B. cereus* strain F4810/72, were traced in the following semi-preparative HPLC-fractions: cereulide (**1**; Fraction 8), isocereulide A (**2**; Fraction 9) (Supplementary Materials, Figure S1B). **1** and **2**, respectively, were then purified from the given fractions via analytical HPLC.

In accordance with the recently reported structure for isocereulide A (**2**) [20], a mass of *m/z* 1184.7281 for the [M+NH_4_]^+^ pseudomolecular ion was observed in UPLC-ESI^+^-TOF-MS measurements, varying +14 Da from the [M+NH_4_]^+^ adduct of cereulide (*m/z* 1170.7125) (Supporting Information, Figure S2, Table S1), resulting in an elemental composition of C_58_H_102_N_7_O_18_, and indicating the addition of one methylene group compared to **1′**s structure.

In comparison to the amino and *α*-hydroxy acid composition of cereulide (**1**) (3 × Ala, 3 × Val, 3 × *O*-Leu, 3 × *O*-Val), one of the amino acids or *α*-hydroxy acids present in the structure of **2** was likely exchanged by a 14 Da heavier homologous unit, as indicated by the correspondingly detected *m/z* for isocereulide A (**2**; *m/z* 1184.7281). Reference [20] suggested an exchange of one l-*O*-Val by one l-*O*-Leu unit, determined by alkaline hydrolysis of the isolated compound to give dipeptide units, followed by a comparison with synthesized dipeptide references. Additionally, further acidic hydrolysis on the gained dipeptide intermediates was performed to yield single amino acids and *α*-hydroxy acids, whereof the amino acids were consequently derivatized and mass-spectrometrically analyzed against treated alike enantiopure references to elucidate their corresponding stereo configuration.

### 2.2. D- and 2D-NMR Spectroscopy of ***2***

**Figure 1:**
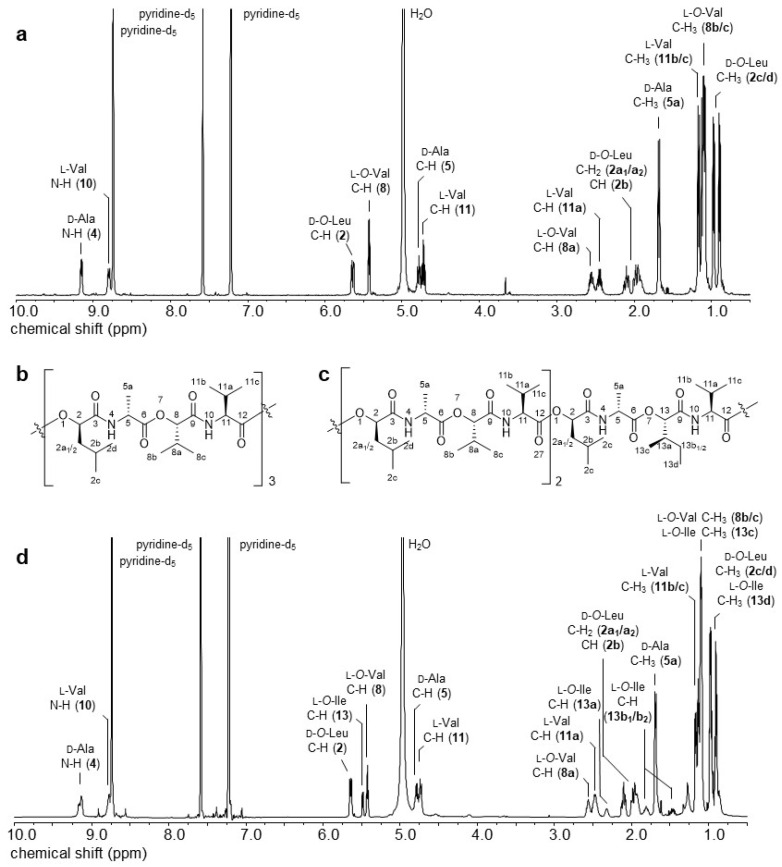
^1^H-NMR spectra and corresponding chemical structures of (**a**,**b**) cereulide (**1**) and (**c**,**d**) isocereulide A (**2**).

The isolation of isocereulide A (**2**) allowed, for the first time, 1D- and 2D-NMR characterization of a natural isocereulide, originating from *B. cereus* samples. In direct comparison to the ^1^H-NMR spectrum of isocereulide A (**2**) (Figure 1d) to the ^1^H-NMR spectrum of cereulide (**1**) (Figure 1a), an exchange of one l-*O*-Val moiety can be concluded, as indicated by the loss of intensity of the corresponding signals (Figure 1d, C-H (8a) δ = 2.55 ppm; C-H (8) δ = 5.43 ppm) and appearance of new proton signals belonging to the substitute *α*-hydroxy acid (Figure 1d, C-H_2_ (13b_1_/b_2_) δ = 1.40 and 1.76 ppm; C-H (13a) δ = 2.30 ppm; C-H (13) δ = 5.48 ppm).

**Figure 2:**
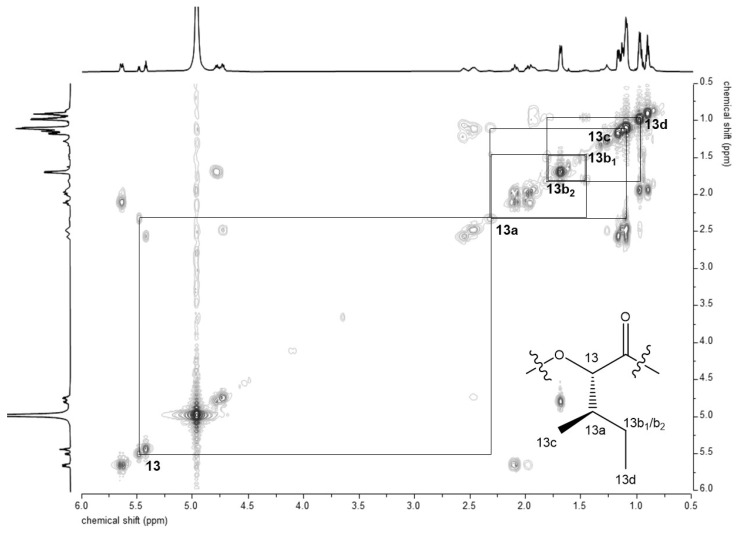
Excerpt of the ^1^H-^1^H-COSY-NMR spectrum with highlighted correlations of the *O*-Ile protons present in the chemical structure of isocereulide A (**2**).

From the further generated ^1^H-^1^H-COSY-spectrum (Figure 2), the correlation of the single proton signals could be observed, giving, in combination with the signals integrals, the structures of the single amino acids Ala and Val (×3 each) and *α*-hydroxy acids *O*-Leu (×3) and *O*-Val (×2), but revealing one additional *O*-Ile moiety. This can be deducted from the correlation of the signals shown in Figure 2, with the key signal being the methine group 13a, exhibiting a correlation to the methyl protons 13c as well as to the methylene protons 13b_1_/b_2_. Those novel NMR experiments shed light on the assumption that in fact the isobaric *α*-hydroxy acid *O*-Ile is present in the chemical structure of **2** instead of the l-*O*-Leu moiety reported up to now [20].

### 2.3. Alkaline Hydrolysis and UPLC-ESI-TOF-MS Analysis of ***2***

**Figure 3:**
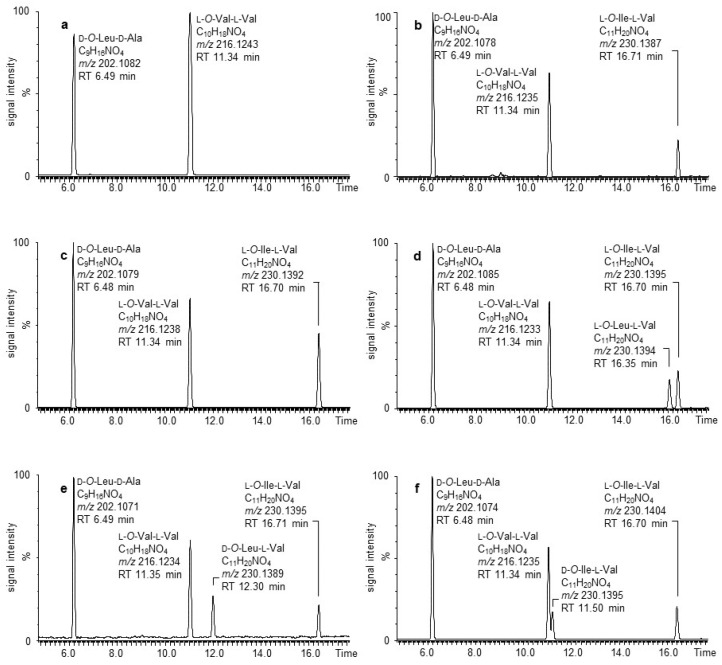
UPLC-ESI^–^-TOF-MS-analyses of alkaline hydrolysates of (**a**) cereulide (**1**), (**b**) isocereulide A (**2**), (**c**) **2** spiked with l-*O*-Ile-l-Val, (**d**) **2** spiked with l-*O*-Leu-l-Val, (**e**) **2** spiked with d-*O*-Leu-l-Val, and (**f**) **2** spiked with d-*O*-Ile-l-Val.

To further support the presence of the *O*-Ile unit in isocereulide A (**2**), the sample material was submitted to alkaline hydrolysis to yield dipeptides, followed by comparison to synthesized reference material and acidic hydrolysis with consequent amino acid and *α*-hydroxy acid derivatization, according to the literature protocol [20,22,23]. After alkaline hydrolysis, **2** revealed in its UPLC-ESI^–^-TOF-MS analysis, next to d-*O*-Leu-d-Ala (*m/z* 202.1088, [M-H]^–^; C_9_H_16_NO_4_) and l-*O*-Val-l-Val (*m/z* 216.1240, [M-H]^–^; C_10_H_18_NO_4_), one additional dipeptide at *m/z* 230.1391 [M-H]^–^, with an empirical elemental composition of C_11_H_20_NO_4_ in a ratio of 3:2:1 (Figure 3b). MS^e^ analysis of the questionable dipeptide highlighted a fragmentation pattern concomitant with both *O*-Leu-Val or *O*-Ile-Val, exhibiting fragment ions at *m/z* 186.1490 ([M-H]^–^; C_10_H_20_NO_2_) and *m/z* 116.0707 ([M-H]^–^; C_5_H_10_NO_2_), respectively (Supporting Information, Figure S4).

By comparing the hydrolysate to the eligible isobaric dipeptides l-*O*-Leu-l-Val, d-*O*-Leu-l-Val, l-*O*-Ile-l-Val, and d-*O*-Ile-l-Val, as well as chromatographic separation of the isomers, the dipeptide at hand was identified (Figure 3). In this chromatographic separation, the l-*O*-Leu-l-Val dipeptide, recently reported to be present in isocereulide A (**2**) [20], showed a retention time shift by 0.35 min compared to the dipeptide naturally present in the alkaline hydrolysis of **2** (Figure 3d). Only the spiking of l-*O*-Ile-l-Val exhibited an increase in the peak intensity of the respective dipeptide and showed no visible additional signal (Figure 3c). Thus, of the aforementioned four isobaric dipeptides, uniquely l-*O*-Ile-l-Val qualified as the dipeptide at hand in the structure of isocereulide A (**2**).

### 2.4. Acidic Hydrolysis and Enantioselective Amino Acid and α-Hydroxy Acid Analysis of ***2***

To unequivocally identify the absolute amino acid and *α*-hydroxy acid composition in isocereulide A (**2**), subsequent acidic hydrolysis, free amino acid analysis according to Reference [20], and, for the first time in an isocereulide, *α*-hydroxy acid analysis were performed. The hydrolysate was analyzed via UPLC-TOF-ESI^–^-MS after enantioselective derivatization of the amino acids and the *α*-hydroxy acids via OPA/IBLC, and *S*-(+)-MTPA chloride, respectively [22,23], followed by a comparison of accurate *m/z* and retention times of treated alike enantiopure amino acid and *α*-hydroxy acid references.

As a reference sample, **1** was treated under the same conditions. For the analysis of isoindole-amino acid derivatives from **2**, fitting well together with the already published data [20], only d- and l-Ala in a ratio of 99.9/0.1 and d- and l-Val in a ratio of 0.1/99.9 could be determined, thus indicating the presence of d-Ala and l-Val as amino acid modules, while l-Ala and d-Val are absent (Supporting Information, Figure S5).

The novel analysis of MTPA-*α*-hydroxy acid esters for **2** revealed the *α*-hydroxy acids d- and l-*O*-Val in a ratio of 0.1/99.9 and d- and l-*O*-Leu and l-*O*-Ile in a ratio of 77.1/0.1/22.8, confirming the presence of l-*O*-Val, d-*O*-Leu, and also l-*O*-Ile while at the same time providing evidence for the lack of d-*O*-Val and l-*O*-Leu. Additionally, the ratio observed for the *α*-hydroxy acids d-*O*-Leu and l-*O*-Ile, is in agreement with the theoretically calculated distribution of 75/25. Thus, the dipeptide units d-*O*-Leu-d-Ala, l-*O*-Val-l-Val, and l-*O*-Ile-l-Val could evidently be identified with a ratio of 3:2:1 as the components present in the chemical structure of isocereulide A (**2**), with l-*O*-Leu-l-Val not occurring within this structure (Supporting Information, Figure S6).

### 2.5. MS^n^ Sequencing of ***2***

**Figure 4:**
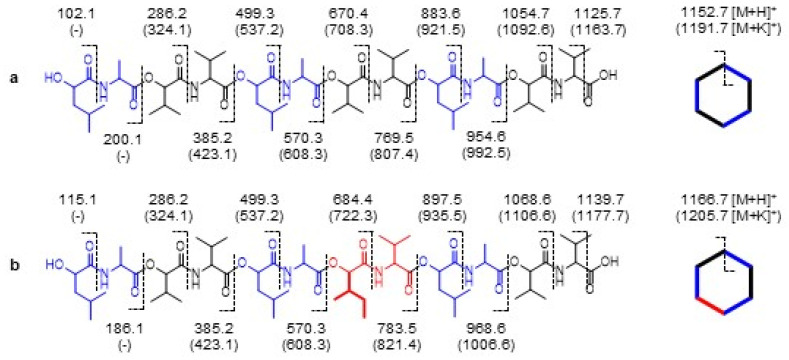
MS^n^ sequences of the selected pseudomolecular ions for the structure elucidation of (**a**) cereulide (**1**) and (**b**) isocereulide A (**2**) (black: l-*O*-Val-l-Val, blue: d-*O*-Leu-d-Ala, red: l-*O*-Ile-l-Val).

Due to multiple theoretically possible arrangements of the identified dipeptide modules within the cyclic dodecadepsipeptide structure of the isocereulide, the exact alignment of the entities was determined by means of sequential MS^n^ fragmentation. The favored ester cleavage of the depsipeptide structure evoked the formation of a maximum of six theoretically possible isobaric open-chain pseudomolecular ions for isocereulide A, resulting from different possible locations of the ring opening. Thereof, one theoretical dipeptide sequence was selected as a precursor for further fragmentation of resulting intermediates according to the selected dipeptide pattern. (Figure 4, MS^n^ data pictured in Supporting Information).

In alignment with the already published data on isocereulide A (**2**) [20], the MS^n^ sequencing revealed the [M+K]^+^ pseudomolecular ions 1205.7 → 1006.6 → 821.4 in the MS, MS^2^, and MS^3^ scans for **2** indicating the elimination of l-*O*-Val-l-Val and d-*O*-Leu-d-Ala. The following MS^4^ scan then exhibited the key fragment (821.4 → 722.3/608.3), giving the location of the l-*O*-Ile-l-Val unit. After that, MS^5^ and MS^6^ exhibited the loss of d-*O*-Leu-d-Ala followed by l-*O*-Val-l-Val showing the fragments of 608.3 → 423.1 → 323.1, thus confirming the general sequence of the to date reported isocereulide A (**2**) [20].

## 3. Materials and Methods

### 3.1. Chemicals and Samples

#### 3.1.1. Chemicals

The following compounds were commercially obtained: chloroform (anhydrous, ≥99%), d-(+)-glucose monohydrate, methanol-*d_4_*, *N*-isobutyryl-l-cysteine (IBLC), *N*,*N*-diisopropylethylamine (DIPEA), *O*-(Benzotriazol-1-yl)-*N*,*N*,*N’*,*N’*-tetraethyluronium hexafluoro phosphate (HBTU), *ortho*-phthaldialdehyde (OPA), piperidine, potassium hydroxide (KOH), potassium tetraborate tetrahydrate (B_4_K_2_O_7_x4H_2_O), pydridine (anhydrous, 99.8%), pyridine-*d_5_*, (*S*)-(–)-2-hydroxyisocaproic acid (l-*O*-Leu), and trifluoroacetic acid (reagent plus, 99%) from Sigma-Aldrich (Steinheim, Germany); Fmoc-l-Val-Wang resin (100-200 mesh each), d- and l-alanine, d- and l-valine, HCl (37%), *N*,*N*-dimethylformamide (DMF), and formic acid (HCOOH) from Merck (Darmstadt, Germany); dichloromethane (CH_2_Cl_2_) from Carl Roth (Karlsruhe, Germany); l-*α*-hydroxyisovaleric acid (l-*O*-Val), d-*α*-hydroxyisovaleric acid (d-*O*-Val), d-*α*-hydroxyisocaproic acid (d-*O*-Leu) from Bachem (Bubendorf, Switzerland); and (2*S*,3*S*)-2-hydroxy-3-methylpentanoic acid (l-*O*-Ile) from Interchim (Montluçon Cedex, France); (2*R*,3*R*)-2-hydroxy-3-methylpentanoic acid (d-*O*-Ile) from SIA Enamine (Riga, Latvia) and (*S*)-(+)-*α*-methoxy-*α*-trifluoromethylphenylacetic acid chloride (MTPA) from TCI Deutschland GmbH (Eschborn, Germany).

H_2_O for chromatography was purified with a Milli-Q Reference A+ System (Merck), solvents used were of HPLC or LC-MS grade (J.T. Baker, Deventer, Holland).

#### 3.1.2. *B. cereus* Culture and Growth Conditions

For the biofermentative generation of cereulide and isocereulide A, cultures of the emetic *B. cereus* strain F4810/72 were prepared as described previously [24]. In brief, 3 mL of lysogeny broth (LB) were inoculated with *B. cereus* strain F4810/72 and incubated for 16 h at 30 °C while shaking (120 rpm). These precultures were used for kinetic inoculation of 100 mL LB medium in rotary flasks with 10^3^ cfu/mL. Cultures were incubated with shaking (120 rpm) at 30 °C for 24 h. After centrifugation (8000 rpm, 2 min) and discarding of the supernatant, the remaining cell pellets were autoclaved (121 °C, 15 min). The pellets were stored at −20 °C until further use.

#### 3.1.3. Solvent Extraction and Toxin Isolation

The pellets of strains F4810/72 were thawed and extracted with EtOH (3 × 30 mL) by shaking for 1 h (400 rpm, RT). The ethanolic extracts were centrifuged (4000 rpm; 10 min), their supernatants membrane filtrated (0.2 µm; PTFE; Phenomenex, Aschaffenburg, Germany) to remove remaining cell material, all liquids combined, and the solvent reduced to one-fifth under low pressure by means of a rotary evaporator. The concentrated ethanolic extract was stored at −20 °C until further use.

The reduced ethanolic extract was diluted with H_2_O (1:10) and prefractionated via C18 SPE cartridges (60 mL, 10 g, Chromabond, Macherey-Nagel, Düren, Germany) according to Reference [20], with preconditioned columns using MeOH (30 mL), MeOH/H_2_O (50/50; *v/v*; 30 mL) and H_2_O (60 mL). To enrich the desired compounds, 15 mL of the prepared cell extract was applied, eluted through the column, and flushed with H_2_O (20 mL). The process was repeated five times. After the final sample application, the columns were rinsed with MeOH/H_2_O (70/30; *v/v*; 60 mL), dried under reduced pressure using a vacuum pump (30 min), and finally, the target molecules eluted with MeOH (60 mL). Obtained methanolic fractions were combined, the solvent removed under reduced pressure via rotary evaporator to approx. 150 mL, and stored at −20 °C until further use for compound isolation.

Semi-preparative HPLC was performed as reported recently [20] on a PrepStar system (Varian, Darmstadt, Germany), consisting of two HPLC pumps (model SD-1), a two-wavelength UV detector (Prostar 325), and a fraction collector (model 701), equipped with a 250 × 10 mm, 4 µm, 90 Å Jupiter Proteo column (Phenomenex, Aschaffenburg, Germany). The effluent was monitored at 220 nm, while chromatography was performed at 4.2 mL/min using H_2_O (Solvent A) and MeOH (Solvent B), starting with 85% B for 1 min, increasing to 92% B in 15 min, holding 92% B for 9 min, increasing to 100% B within 1 min, holding 100% B for 9 min, and decreasing to 85% B in 1 min, followed by equilibration for 2 min. The eluate was separated into 10 fractions (Supporting Information; Figure S1B), and respective liquids were combined. After solvent removal by using a rotary evaporator, water (15 mL) was added, and the fractions were freeze-dried twice and stored until further fractionation at −20 °C.

The obtained HPLC-fractions were screened for cereulide (**1**) and the isocereulides A–G by means of an UPLC-TOF-MS system Synapt G2-S (Waters, Manchester, UK) in the positive electrospray mode equipped with a 2.1 × 150 mm, 1.7 µm, BEH-C18 column (Waters, Manchester, UK) at a flowrate of 0.4 mL/min at 45 °C using aqueous HCOOH (0.1%) as Solvent A and MeCN with HCOOH (0.1%) as Solvent B. Elution was started at 93% B and increased to 100% B in 4 min, held at 100% B for 2 min, followed by decreasing to 93% in 0.1 min, and holding at 93% B for 0.9 min.

Analytical purification was performed on an HPLC system (Jasco, Groß-Umstadt, Germany) consisting of an HPLC-pump (PU 2080 Plus), a degasser (DG-2080-53 3-Line-Degasser), a DAD/UV detector (MD-2010 Plus), coupled with an autosampler (AS-2055 Plus) and equipped with a 250 × 4.6 mm, Jupiter^®^ 4 µm Proteo 90Å (Phenomenex, Aschaffenburg, Germany). Chromatography was performed at 1 mL/min with H_2_O (Solvent A) and MeOH (Solvent B), starting at 80% B for 3 min, increasing to 92% B within 10 min, holding isocratically for 17 min, increasing to 100% B within 0.5 min and staying at 100% B for 1.5 min, decreasing to 80% B within 0.5 min and finally equilibrating at 80% B for 1.5 min. The effluent was monitored at 220 nm, and eluting substances were collected manually.

#### 3.1.4. Analysis of Dipeptides, Resulting of Alkaline Hydrolysis of **1** and **2**

Following Reference [20], the purified cereulide (**1**) and isocereulide A (**2**) (~500 µg), respectively, were dissolved in methanolic KOH (1.2 M, 80% MeOH), heated to 50 °C for 2 h. The pH value was adjusted to pH 5.0, and the hydrolysates were applied for UPLC-TOF-MS measurement on a Synapt G2-S system (Waters, Manchester, UK), using the spectrometric parameters in the negative electrospray ionization mode, as reported recently [20]. Chromatography was performed on a 2.1 × 150 mm, 1.7 µm, BEH-C18 column (Waters, Manchester, UK), using aqueous HCOOH (0.1%) as Solvent A and a mixture of MeCN/HCOOH (99.9/0.1; *v/v*) as Solvent B at a flowrate of 0.4 mL/min at 45 °C, started at 10% B for 1 min, increased to 20% B in 14 min, to 30% B in 5 min, and to 100% B in 0.5 min. It was then kept at 100% B for 1 min, decreased to 10% B within 0.5 min, and equilibrated at 10% B for 1 min.

#### 3.1.5. Acidic Hydrolysis and Analysis of Amino Acids as well as α-Hydroxy Acids

To determine the stereochemistry of the single amino acids and *α*-hydroxy acids contained in the analyzed dipeptides forming the isocereulide structure, aliquots (~200 µL) of the alkaline hydrolysates of **1** and **2**, respectively, were further used for acidic hydrolysis (3 mL HCl, 6 M, 24 h, 110 °C), adjusted to pH 7.0 with KOH (1 M), and freeze-dried [20]. The lyophilisates were extracted with MeCN (5 mL), centrifuged (4200 rpm, 10 min), and separated into the amino acid containing the salt residue and the *α*-hydroxy acid containing liquid phase. Remaining MeCN was evaporated under a stream of nitrogen. In the following, both aliquots of the acidic hydrolysate of **1** and **2** were put to enantioselective derivatization. Therefore, the derivatization of the amino acids in cereulide (**1**) and isocereulide A (**2**), as well as the corresponding reference amino acids d/l-Ala and d/l-Val (50 µmol each), was performed using OPA and IBLC [22] followed by SPE purification considering Reference [20]. The methanolic eluate gained via SPE was analyzed via UPLC-TOF-MS, applying the same mass spectrometric parameters as described for the alkaline hydrolysis. Chromatography was performed at 45 °C and a flowrate of 0.4 mL/min on a 2.1 × 150 mm, 1.7 µm, BEH-C18 column (Waters, Manchester, UK) with aqueous HCOOH (0.1%) as Solvent A, and a mixture of MeCN/HCOOH (99.9/0.1; *v/v*) as Solvent B, while starting the gradient at 30% B for 3 min, increasing to 33% B in 3 min, to 50% B in 6 min, and to 100% B in 1 min, followed by holding 100% B for 1 min, decreasing to 30% B within 0.5 min, and ending with an equilibration at 30% B for 1.5 min. The mass spectrometric data, obtained during the analysis of the amino acid isoindole derivatives, was summarized in the following: d-Ala derivative: accurate mass: *m/z* 377.1180, Δ (ppm): +2.4; calcd: *m/z* 377.1171 (C_18_H_21_N_2_O_5_S); l-Ala derivative: accurate mass: *m/z* 377.1181, Δ (ppm): +2.7; calcd: *m/z* 377.1171 (C_18_H_21_N_2_O_5_S); d-Val derivative: accurate mass: *m/z* 405.1489, Δ (ppm): +1.2; calcd: *m/z* 405.1484 (C_20_H_25_N_2_O_5_S); l-Val derivative: accurate mass: *m/z* 405.1492, Δ (ppm): +2.0; calcd: *m/z* 405.1484 (C_20_H_25_N_2_O_5_S).

Enantioselective derivatization of *α*-hydroxy acids was performed applying *S*-(+)-MTPA chloride, in accordance with Reference [23]. Pydridine (anhydrous, 1 µL) was added to enantiomeric pure reference acids d/l-*O*-Val, d/l-*O*-Leu, and d/l-*O*-Ile (~0.5 µmol each) and the *α*-hydroxy acid containing hydrolysate layer of **1** and **2**, respectively, followed by dissolution of the sample material in anhydrous chloroform (100 µL), supplementing *S*-(+)-MTPA chloride (1 µL), and stirring for 1 h at room temperature. The solution was diluted 1:100 with MeCN and applied for UPLC-TOF-MS experiments. MS parameters were identical to those described for the analysis of amino acids with the following solvent gradient: start at 30% B and hold for 1 min, increase to 50% B in 10 min, to 53% B within 5 min, and to 100% B in 1 min, hold at 100% B for 1 min, decrease in 0.5 min to 30% B, and hold for 1.5 min. The analytical data obtained for the individual *α*-hydroxy acid MTPA esters were the following: d-*O*-Val derivative: accurate mass: *m/z* 333.0949, Δ (ppm): −0.3; calcd: *m/z* 333.0950 (C_15_H_16_F_3_O_5_); l-*O*-Val derivative: accurate mass: *m/z* 333.0950, Δ (ppm): 0.0; calcd: *m/z* 333.0950 (C_15_H_16_F_3_O_5_); d-*O*-Leu derivative: accurate mass: *m/z* 347.1106, Δ (ppm): 0.0; calcd: *m/z* 347.1106 (C_16_H_18_F_3_O_5_); l-*O*-Leu derivative: accurate mass: *m/z* 347.1108, Δ (ppm): + 0.6; calcd: *m/z* 347.1106 (C_16_H_18_F_3_O_5_); d-*O*-Ile derivative: accurate mass: *m/z* 347.1106, Δ (ppm): 0.0; calcd: *m/z* 347.1106 (C_16_H_18_F_3_O_5_); l-*O*-Ile derivative: accurate mass: *m/z* 347.1107, Δ (ppm): + 0.3; calcd: *m/z* 347.1106 (C_16_H_18_F_3_O_5_).

### 3.2. Technical Data

#### 3.2.1. Mass Spectrometry

High-resolution mass spectrometry (UPLC-ESI-TOF-MS) was performed on a Waters Synapt G2-S HDMS spectrometer combined with an Acquity UPLC core system (Waters, Manchester, UK), composed of a binary solvent manager, sample manager, and column. For system operation and data processing, the MassLynx 4.1 SCN 851 Software (Waters, Manchester, UK) was exercised. All data were corrected via lock mass referencing on the pentapeptide leucine enkephalin (Tyr-Gly-Gly-Phe-Leu, *m/z* 556.2771, [M+H]+ and 554.2615, [M−H]-), which was supplied as 2 ng/µL solved in MeCN/0.1% HCOOH (1/1, *v/v*). Scan time for lock mass was set to 0.3 s, an interval of 10 s, and 3 scans to average, with a mass window of ± 0.5 Da. Calibration of the Synapt G2-S was performed in the range from *m/z* 50 to 1200 with a solution of sodium formate (5 mmol/L) in 2-propanol/H_2_O (9/1, *v/v*). Source parameters were applied according to Reference [20].

MS^n^ measurements were carried out on a Bruker Daltonics HCTultra PTM Discovery System (Bruker Daltonics Billerica, MA, USA), following the literature protocol [20].

#### 3.2.2. Nuclear Magnetic Resonance Spectroscopy

**Figure 5:**
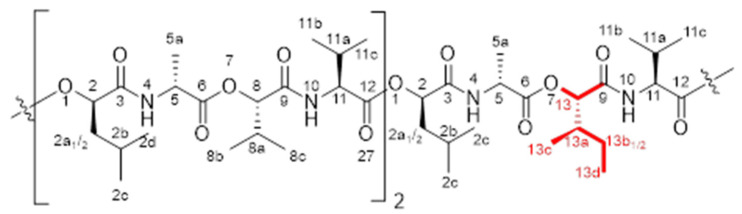
Open-chain chemical structure of isocereulide A (2).

Nuclear magnetic resonance (NMR) spectra were recorded using a 400 MHz Avance III spectrometer with a Broadband Observe BBFO plus and a 500 MHz Avance NEO spectrometer with a cryoprobe CTCI (^1^H/^13^C/^15^N) (Bruker, Rheinstetten, Germany). The chemical shift was referenced to the solvent signal, MeOH-*d_4_* and pyridine-*d*_5_, respectively. Data processing and evaluation were performed using the Topspin Software Version 4.0.7 (Bruker, Rheinstetten, Germany) (Figure 5).

^1^H-NMR, COSY [500 MHz, pyridine-*d*_5_, 298 K]: δ (ppm) 0.90 [d, 9H, *J* = 6.18 Hz, H_3_-C(2c)], 0.97 [m, 12H, H_3_-C(2d, 13d)], 1.07–1.22 [m, 33H, H_3_-C(8b, 8c, 11b, 11c, 13c)], 1.38–1.50 [m, 1H, H-C(13b_1_)], 1.68 [dd, 9H, *J* = 6.2 Hz, H-C(5a)], 1.72-1.83 [m, 1H, H-C(13b_2_)], 1.88–2.03 [m, 6H, H-C(2a_1_, 2b)], 2.06–2.16 [m, 3H, H-C(2a_2_)], 2.27–2.33 (m, 1H, H-C(13a)], 2.38–2.50 [m, 3H, H-C(11a)], 2.50–2.61 [m, 2H, H-C(8a)], 4.69–4.75 [dd, 3H, *J* = 7.5, 15.2 Hz, H-C(11)], 4.75-4.82 [quint, 3H, *J* = 6.6, 13.3 Hz, H-C(5)], 5.43 [dd, 2H, *J* = 4.6, 8.9 Hz, H-C(8)], 5.48 [d, 1H, *J* = 5.0 Hz, H-C(13)], 5.62–5.67 [m, 3H, H-C(2)], 8.76–8.80 [m, 3H, H-N(10)], 9.15 [dd, 3H, H-N(4)]. DEPT135, HSQC, HMBC [125 MHz, pyridin-*d*_5_, 298 K]: δ (ppm) 11.6 [C(13d)], 15.5 [C(13c)], 17.18 [C(5a)], 17.24 [C(13b)], 17.5 [C(8b)], 19.06/19.10/19.5 [C(8c/11b/11c)], 21.4 [C(2c)], 23.4 [C(2d)], 24.79 [C(2b)], 24.84 [C(13b)], 30.0 [C(11a)], 30.4 [C(11a)], 30.5 C[(11a)], 31.2 [C(8a)], 37.4 [C(13a)], 41.4 [C(2a)], 49.71 [C(5)], 49.74 [C(5)], 49.77 [C(5)], 59.18 [C(11)], 59.25 [C(11)], 59.28 [C(11)], 73.3 [C(2)], 78.5 [C(13)], 79.0 [C(8)], 170.4 [C(9)], 170.6 [C(9)], 170.9 [C(9)], 171.1 [C(3)], 171.26 [C(3)], 171.34 [C(3)].

## 4. Conclusions

The emetic *B. cereus* toxin cereulide and its isocereulides comprise a complex, three-dimensional dodecadepsipeptide structure [5,6,7,8]. For the to date structurally known isocereulides A–E, one amino acid or *α*-hydroxy acid is exchanged, while for isocereulides G and F a dipeptide section is switched or replaced. Isocereulide A in particular was reported as [(d-*O*-Leu-d-Ala-l-*O*-Val-l-Val)_2_(d-*O*-Leu-d-Ala-l-*O*-Leu-l-Val)] beforehand [20], differing from cereulide only by the exchange of one l-*O*-Val unit by one l-*O*-Leu moiety. With the investigation of isocereulide A in this study, for the first time, 1D- and 2D-NMR techniques were employed to investigate a natural isocereulide and by additional UPLC-ESI-TOF-MS dipeptide, amino acid and *α*-hydroxy acid analysis, and MS^n^ sequencing, the chemical structure of isocereulide A was revised to [(d-*O*-Leu-d-Ala-l-*O*-Val-l-Val)_2_(d-*O*-Leu-d-Ala-l-*O*-Ile-l-Val)].

## Data Availability

The data presented in this study are available in the Supporting Information.

## Supplementary Materials

The following are available online at, **Figure S1A:** Cyclic structure of cereulide. **Figure S1B:** Chromatogram of the semi-preparative RP-HPLC separation of the ethanolic extract of *B. cereus* strain F4810/72, and analytical RP-HPLC run of Fraction 9 for isolation of isocereulide A (**2**). **Figure S2:** UPLC-ESI^+^-TOF-MS measurements of (**a**) the newly isolated isocereulide A (**2**) and (**b**) reference material of isocereulide A (**2**), obtained from [20]. **Table S1:** UPLC-ESI^–^-TOF-MS data of cereulide (**1**) and isocereulide A (**2**). **Figure S3**: Structures of synthesized reference dipeptide units, with numbered atoms for assignment of NMR data. **Figure S4:** Mass-spectrometric fragmentation pattern (UPLC-ESI-TOF-MS^e^) of dipeptides (**a**) d-*O*-Leu-d-Ala, and (**b**) l-*O*-Val-l-Val present in cereulide (**1**), (**c**) l-*O*-Leu-l-Val in the predicted structure for isocereulide A [20], (**d**) and l-*O*-Ile-l-Val present in the newly elucidated structure of isocereulide A (**2**). **Figure S5:** UPLC-ESI^–^-TOF-MS chromatograms of acidic hydrolysis after chiral amino acid derivatization of (**a**) amino acid references l- and d-alanine and l- and d-valine, (**b**) cereulide (**1**), and (**c**) isocereulide A (**2**). **Figure S6:** UPLC-ESI^–^-TOF-MS chromatograms of acidic hydrolysis after chiral *α*-hydroxy acid derivatization of (**a**) *α*-hydroxy acid references l- and d-*O*-valine, and l- and d-*O*-leucine with l- and d-*O*-isoleucine, (**b**) cereulide (**2**), (**c**) isocereulide A (**1**). **Figure S7:** ^1^H-NMR spectrum of **1** (500 MHz, 298 K, Pyridine-d_5_). **Figure S8:** ^1^H,^1^H-COSY-NMR spectrum of **1** (500 MHz, 298 K, Pyridine-d_5_). **Figure S9:** ^1^H,^13^C-HSQC-NMR spectrum of **1** (500 MHz, 125 MHz, 298 K, Pyridine-d_5_). **Figure S10:** ^1^H,^13^C-HMBC-NMR spectrum of **1** (500 MHz, 125 MHz, 298 K, Pyridine-d_5_). **Figure S11:** ^13^C-NMR spectrum of **1** (125 MHz, 298 K, Pyridine-d_5_). **Figure S12:** ^1^H-NMR spectrum of **2** (500 MHz, 298 K, Pyridine-d_5_). **Figure S13A:** ^1^H,^1^H-COSY-NMR spectrum of **2** (500 MHz, 298 K, Pyridine-d_5_). **Figure S13B:** Zoom ^1^H,^1^H-COSY-NMR spectrum of **2** (500 MHz, 298 K, Pyridine-d_5_). **Figure S14:** ^1^H,^13^C-HSQC-NMR spectrum of **2** (500 MHz, 125 MHz, 298 K, Pyridine-d_5_). **Figure S15A:** ^1^H,^13^C-HMBC-NMR spectrum of **2** (500 MHz, 125 MHz, 298 K, Pyridine-d_5_). **Figure S15B:** Zoom ^1^H,^13^C-HMBC-NMR spectrum of **2** (500 MHz, 125 MHz, 298 K, Pyridine-d_5_). **Figure S16:** DEPT135-NMR spectrum of **2** (125 MHz, 298 K, Pyridine-d_5_). Additionally, data on synthesis, purification, and NMR characterization of dipeptides and data on MS^n^ sequencing are available.

## Sample Availability

Sample of the compound cereulide is available from the authors.
