# Supplementary material for: Structure Revision of Isocereulide A, an Isoform of the Food Poisoning Emetic Bacillus cereus Toxin Cereulide
Source: Molecules. 2021 Mar 4;26(5):1360. doi: 10.3390/molecules26051360 (PMC7961578; doi:10.3390/molecules26051360)
Supplement: Supplementary file 1 [file molecules-26-01360-s001.pdf]

molecules

MDPI

Supplementary Items

Structure revision of isocereulide A, an isoform of the food poisoning emetic *B. cereus* toxin cereulide

Veronika Walser†, Markus Kranzler‡, Monika Ehling-Schulz‡, Timo D. Stark† and Thomas F. Hofmann†

† Food Chemistry and Molecular Sensory Science, Technical University of Munich, Lise-Meitner-Str. 34, 85354 Freising, Germany,
‡ Institute of Microbiology, Department of Pathobiology, University of Veterinary Medicine Vienna, Veterinärplatz 1, 1210 Vienna, Austria.

Isolation of cereulide (1) and isocereulide A (2) *via* RP-HPLC

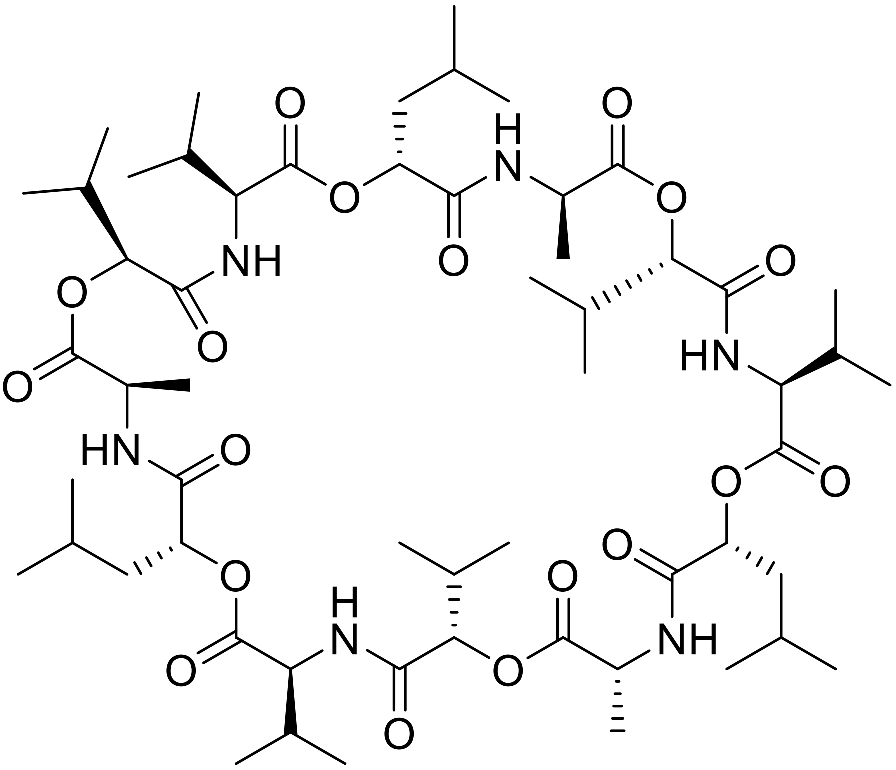

Figure 1. A: Cyclic structure of cereulide (**1**).

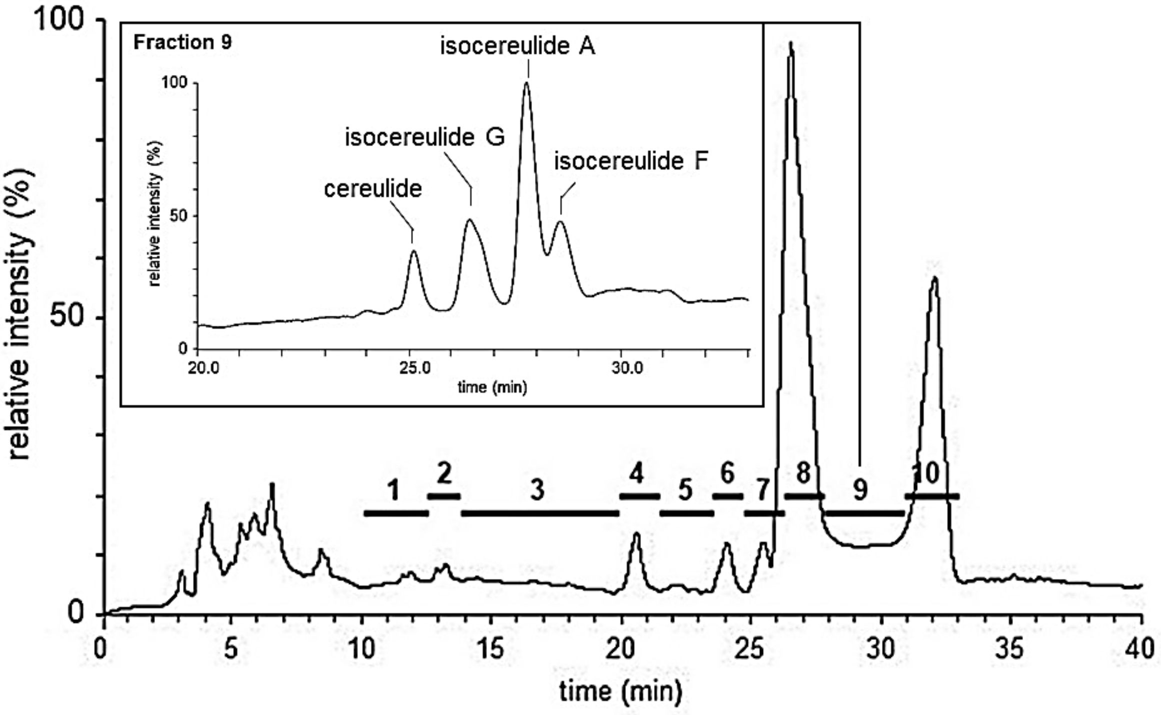

Figure 1. B: Chromatogram of the semi-preparative RP-HPLC separation of the ethanolic extract of *B. cereus* strain F4810/72, and analytical RP-HPLC run of fraction 9 for isolation of isocereulide A (**2**).

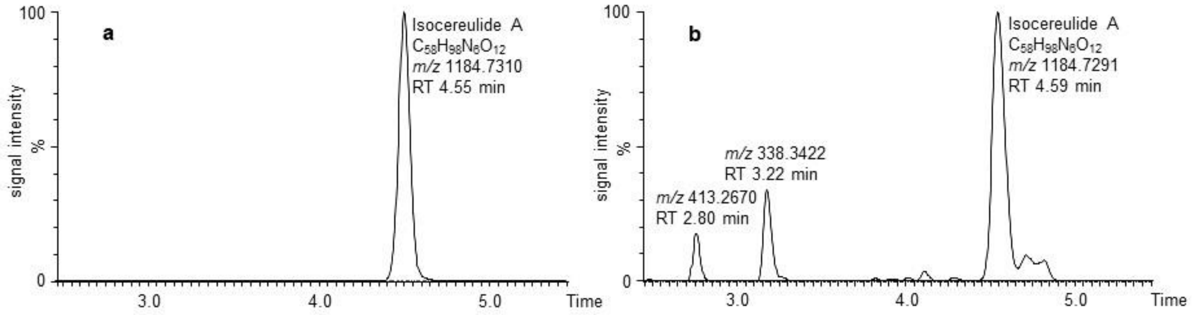

Figure 2. UPLC-ESI$^+$-TOF-MS measurements of (**a**) the newly isolated isocereulide A (**2**) and (**b**) reference material of isocereulide A (**2**) obtained from Marxen *et al.* (2015).

Table 1. UPLC-ESI$^-$-TOF-MS data of cereulide (**1**) and isocereulide A (**2**).

**No.**[a]	Variant[b]	F no.[c]	UPLC-ESI+-TOD-MS data			Structural modification[h]	
			RT[d] (min)	EM/AM[e] (var., ppm)	EC[f] ⊚mass[g] (Da)	Cereulide	Variant
**1**	Cereulide	8	4.01	1170.7125/1170.7161 (+3.1)	$C_{57}H_{100}N_7O_{18}$ (–)	–	–
**–**	Isocereulide A (Marxen *et al.* 2015 [1])	–	4.59	1184.7281/1184.7291 (+0.8)	$C_{58}H_{102}N_7O_{18}$ (+14)	L-*O*-Val	L-*O*-Leu
**2**	Isocereulide A	9	4.55	1184.7281/1184.7310 (+2.4)	$C_{58}H_{102}N_7O_{18}$ (+14)	L-*O*-Val	L-*O*-Ile

[a] Compound number of detected cereulide variant in order of reference in the text; [b] Structures of cereulide and isocereulide A are pictured in **Figure 4.** Reference substance for isocereulide A was obtained from Marxen *et al.* (2015) [1]; [c] HPLC fraction of *B. cereus* strain culture extract F4810/72 used for isolation of target compounds. Numbers of fractions are given according to the semi-preparative HPLC-fractionation shown in **Figure S1**; [d] Retention time on RP-C18 UPLC; [e] Exact mass (EM), calculated from elemental composition, and accurate mass (AM) of pseudo molecular ions $[M+NH_4]^+$ of analytes determined via UPLC-ESI$^+$-TOF-MS; [f] Elemental composition of the analyte; [g] Mass difference between cereulide and the target variant; [h] Listed α-hydroxy acids in cereulide are replaced by the ones enlisted for each variant

Dipeptide synthesis, isolation and characterization *via* 1D- and 2D-NMR data and MS^e fragmentation

For the synthesis of adequate dipeptide references, an aliquot of enantiomeric pure wang resin bound Fmoc-L-Val (0.1 mmol), was steeped in DMF for 30 minutes [1–3]. After washing the resin with DMF (3 x 5 ml), piperidine in DMF (3 x 3 ml, 20 %) was added for a reaction time of 2 minutes, followed by another washing step with DMF (3 x 5 ml). Separately, *N,N*-Diisopropylethylamine (1.0 mmol) was added to a solution of *O*-(benzotriazol-1-yl)-*N,N,N′,N′*-tetramethyluronium hexafluorophosphate (0.49 mmol) and (2*R*,3*R*)-2-hydroxy-3-methylpentanoic acid (D-*O*-Ile), and (2*S*,3*S*)-2-hydroxy-3-methylpentanoic acid (L-*O*-Ile) (0.5 mmol), respectively, in DMF (1 ml). While implementing a nitrogen atmosphere, the solution was mixed with the prepared wang resin and left for reaction at room temperature for 1 h. Thereafter, the resin was washed with dichloromethane (5 x 3 ml), methanol (5 x 3 ml) and the resin bound dipeptide released under nitrogen atmosphere by applying a mixture of trifluoroacetic acid and water (95/5, v/v, 5 ml) and stirring overnight. Subsequently, the solved dipeptide was separated from the resin *via* filtration and washing the residue with a mixture of trifluoroacetic acid and water (95/5, v/v, 2 x 2 ml), followed by water (2 x 2 ml). After unifying all liquids, excess water and acid were removed by freeze drying twice.

Purification of the dipeptides was obtained via semi-preparative HPLC, consisting of two PU-2087 pumps (Jasco, Groß-Umstadt, Germany), DG-2080-53 degasser (Jasco, Groß-Umstadt, Germany), and UV 2075 detector (Jasco, Groß-Umstadt, Germany). The sample was manually injected using a Rh 7725i loop injection valve (Rheodyne, Bensheim, Germany), data evaluation was performed *via* Chrompass 1.8.6.1 (Jasco, Groß-Umstadt, Germany). The column was kept at room temperature with solvent A being 0.1 % aqueous HCOOH, and solvent B being MeCN (0.1 % HCOOH), at a flow of 4 ml/min. The effluent was monitored at 220 nm. The specific parameters for the dipeptides D-*O*-Ile-L-Val and L-*O*-Ile-L-Val are as follows:

Stationary phase: 250 × 10 mm, Synergi 4u Polar-RP 80A (Phenomenex, Aschaffenburg, Germany)

Gradient: 30 % B isocratically for 3 min, increase in 7 min to 70 % B, increase in 2 min to 100 % B, hold for 2 min, decrease within 1 min to 30 % B, followed by 3 min equilibration time.

The solvent of the purified dipeptides (D-*O*-Ile-L-Val, L-*O*-Ile-L-Val) was evaporated under nitrogen current and the substance suspended in water (5 ml). After freeze drying, molecular characterization and structure determination was performed by applying 1D- and 2D-NMR-spectroscopy and UPLC-TOF-MS^e experiments. The dipeptides D-*O*-Leu-D-Ala, D-*O*-Leu-L-Val, L-*O*-Leu-L-Val and L-*O*-Val-L-Val were obtained by Sandra Marxen [1]. NMR-data for L-*O*-Leu-L-Val and D-*O*-Leu-L-Val are according to literature [1]. The chemical structures and NMR-data of all dipeptides are enlisted below, the numeration of single atoms is corresponding to **Figure S3**.

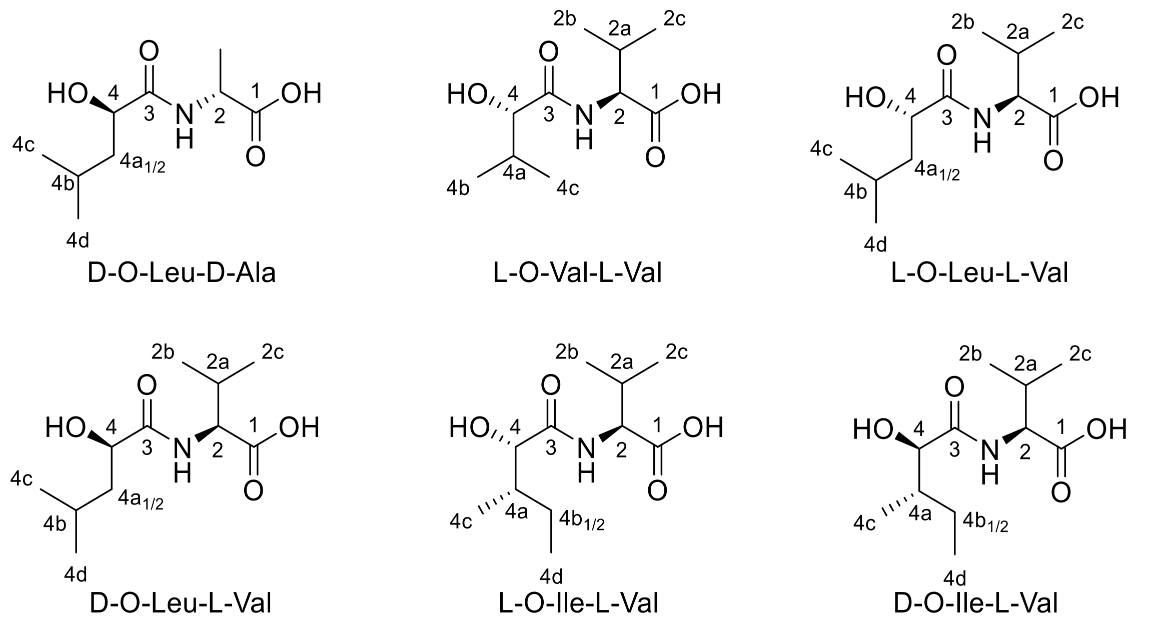

Figure 3. Structures of synthesized reference dipeptide units, with numbered atoms for assignment of NMR data.

D-*O*-Leu-D-Ala: ^{1}H-NMR [500 MHz, d_3-MeOD, COSY, 298 K]: ◎◎ 0.95 [d, 6H, *J* = 6.7 Hz, H-C(4c, d)], 1.41 [d, 3H, *J* = 7.3 Hz, H-C(2a)], 1.44 – 1.59 [m, 2H, H-C(4a)], 1.81 – 1.91 [m, 1H, H-C(4b)], 4.05 [dd, 1H, *J* = 3.7, 9.6 Hz, H-C(4)], 4.38 [q, 1H, *J* = 7.2 Hz, H-C(2)]. ^{13}C-NMR [125 MHz, d_3-MeOD, HSQC, HMBC, 298 K]: δ 18.53 [C (2a)], 21.92 [C (4c)], 24.09 [C (4d)], 25.65 [C (4b)], 44.85 [C (4a)], 49.32 [C (2)], 71.42 [C (4)], 176.62 [C (1)], 177.55 [C (3)].

L-*O*-Val-L-Val: ^{1}H-NMR [400 MHz, d_3-MeOD, COSY, 298 K]: δ 0.87 [d, 3H, *J* = 6.8 Hz, H_3-C (4b)], 0.96 [d, 3H, *J* = 7.1 Hz, H_3-C (2b)], 0.98 [d, 3H, *J* = 7.1 Hz, H_3-C (2c)], 1.02 [d, 3H, *J* = 7.0 Hz, H_3-C (4c)], 2.05 – 2.16 [m, 1H, H-C (4a)], 2.16 – 2.28 [m, 1H, H-C (2a)], 3.89 [d, 1H, *J* = 3.4 Hz, H-C (4)], 4.36 [d, 1H, *J* = 4.7 Hz, H-C (2)]. ^{13}C-NMR [100 MHz, d_3-MeOD, HSQC, HMBC, 298 K]: δ 16.39 [C (4b)], 18.23 [C (2b)], 19.82 [C (2c, 4c)], 32.33 [C (2a)], 33.01 [C (4a)], 58.62 [C (2)], 77.22 [C (4)], 175.05 [C (1)], 176.57 [C (3)].

L-*O*-Leu-L-Val: ^{1}H NMR [500 MHz, d_3-MeOD, COSY, 298 K]: δ 0.96 [m, 12H, H-C(2b,2c, 4c, 4d)], 1.53 [m, 2H, H-C(4a)], 1.87 [m, 1H, H-C(4b)], 2.20 [m, 1H, H-C(2a)], 4.07 [dd, 1H, *J* = 9.7, 3.5 Hz, H-C(4)], 4.34 [d, 1H, *J* = 5.0 Hz, H-C(2)]. ^{13}C NMR [125 MHz, d_3-MeOD, HMBC, HSQC, 298 K]: δ 18.0 [C(2b, 2c)], 19.6 [C(2b,2c)], 21.7 [C(4c,4d)], 24.0 [C(4c, 4d)], 25.6 [C(4b)], 32.3 [C(2a)], 45.0 [C(4a)], 58.5 [C(2)], 71.5 [C(4)], 175.2 [C(1)], 177.6 [C(3)].

D-*O*-Leu-L-Val: ^{1}H NMR [500 MHz, d_3-MeOD, COSY, 298 K]: δ 0.96 [m, 12H, H-C(2b,2c, 4c, 4d)], 1.53 [m, 2H, H-C(4a)], 1.86 [m, 1H, H-C(4b)], 2.21 [m, 1H, H-C(2a)], 4.10 [dd, 1H, *J* = 4.3, 8.9 Hz, H-C(4)], 4.34 [d, 1H, *J* = 5.1 Hz, H-C(2)]. ^{13}C NMR [125 MHz, d_3-MeOD, HMBC, HSQC, 298 K]: δ 18.1 [C(2b, 2c)], 19.5 [C(2b,2c)], 21.8 [C(4c,4d)], 23.9 [C(4c, 4d)], 25.5 [C(4b)], 32.1 [C(2a)], 44.6 [C(4a)], 58.4 [C(2)], 71.4 [C(4)], 174.6 [C(1)], 177.7 [C(3)].

L-*O*-Ile-L-Val: ^{1}H-NMR [500 MHz, d_3-MeOD, COSY, 298 K]: ◎ 0.83 [t, 3H, *J* = 7.4 Hz, H-C(4d)], 0.87 [d, 3H, *J* = 6.8 Hz, H-C(2b)], 0.89 [d, 3H, *J* = 6.8 Hz, H-C(2c)], 0.93 [d, 3H, *J* = 6.9 Hz, H-C(4c)], 1.17 – 1.32 [m, 1H, H-C(4b_1)], 1.39 – 1.52 [m, 1H, H-C(4b_2)], 1.79 – 1.90 [m, 1H, H-C(4a)], 2.16 – 2.27 [m, 1H, H-C(2a)], 3.85 [d, 1H, *J* = 3.6 Hz, H-C(4)], 4.22 [d, 1H, *J* = 3.6 Hz, H-C(2)]. ^{13}C-NMR [125 MHz, d_3-MeOD, HSQC, HMBC, 298 K]: δ 12.40 [C (4d)], 16.28 [C (4c)], 18.35 [C (2b)], 20.21 [C (2c)], 24.66 [C (4b)], 32.70 [C (2a)], 39.90 [C (4a)], 60.00 [C (2)], 77.43 [C (4)], 176.36 [C (3)], 177.10[C (1)].

D-*O*-Ile-L-Val: ^{1}H-NMR [500 MHz, d_3-MeOD, COSY, 298 K]: δ 0.79 [d, 3H, *J* = 6.9 Hz, H_3-C(4b)], 0.88 – 0.99 [m, 9H, H_3-C(2b,2c,4d)], 1.25 – 1.54 [m, 2H, H_2-C(4c)], 1.81 [hd, 1H, *J* = 7.0, 2.8 Hz, H-C(4a)], 2.19 [hd, 1H, *J* = 6.9, 5.1 Hz, H-C(2a)], 4.04 [d, 1H, *J* = 2.8 Hz, H-C(4)], 4.32 [d, 1H, *J* = 5.1 Hz, H-C(2)]. ^{13}C-NMR [125 MHz, d_3-MeOD, HMBC, HSQC, 298 K]: δ 12.19 [C(4d)], 13.38 [C(4b)], 18.08 [C(2b)], 19.61 [C(2c)], 27.35 [C(4c)], 32.16 [C(2a)], 39.64 [C(4a)], 58.49 [C(2)], 74.68 [C(4)], 174.85 [C(1)], 177.05 [C(3)].

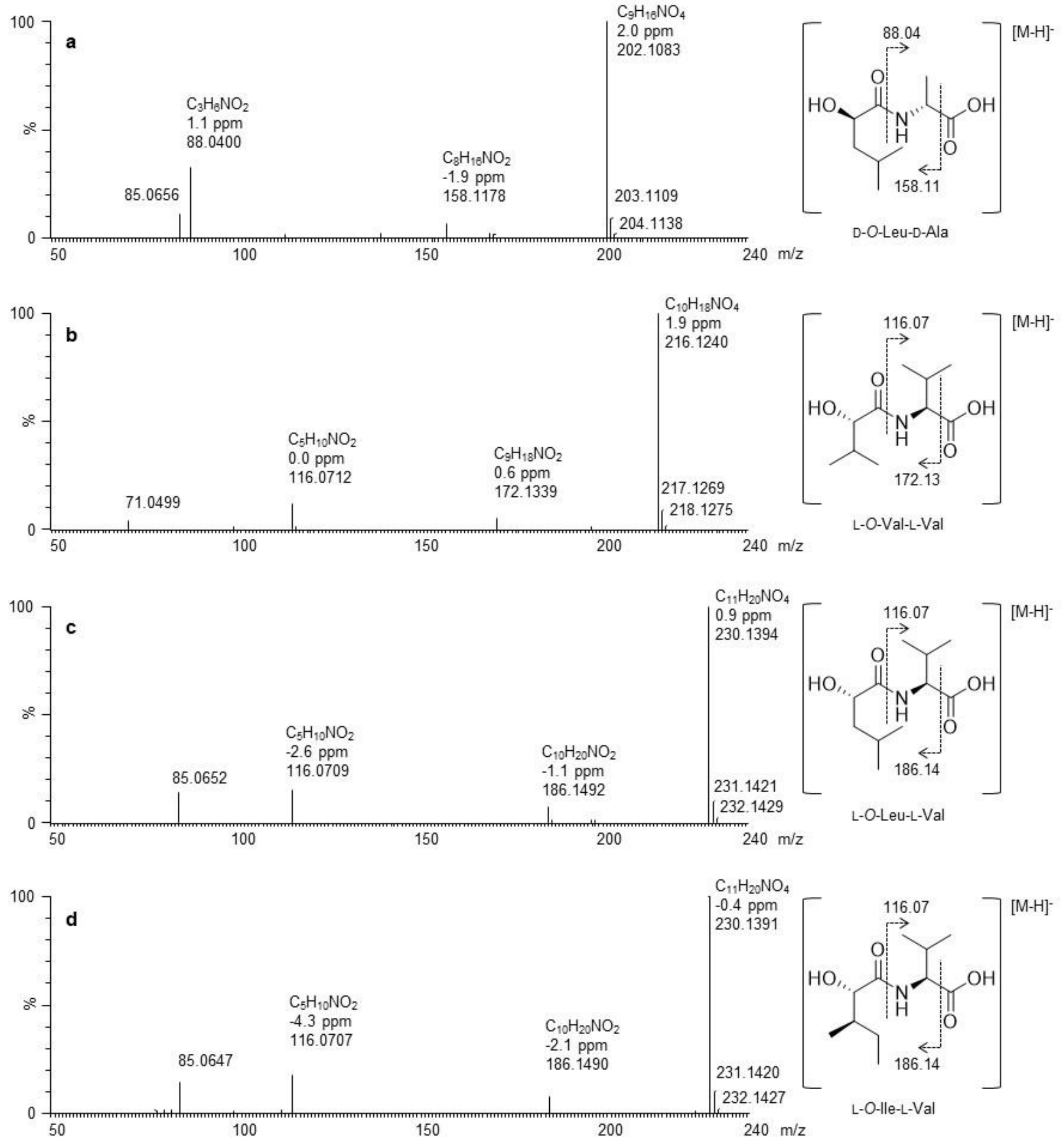

Figure 4. Mass-spectrometric fragmentation pattern (UPLC-ESI-TOF-MS^e) of dipeptides (**a**) D-*O*-Leu-D-Ala, and (**b**) L-*O*-Val-L-Val present in cereulide (**1**), (**c**) L-*O*-Leu-L-Val in the predicted structure for isocereulide A [1] (**d**) and L-*O*-Ile-L-Val, present in the newly elucidated structure of isocereulide A (**2**).

Enantioselective amino acid and α-hydroxy acid analysis in acidic hydrolysates of cereulide (**2**) and isocereulide A (**1**), after chiral derivatization

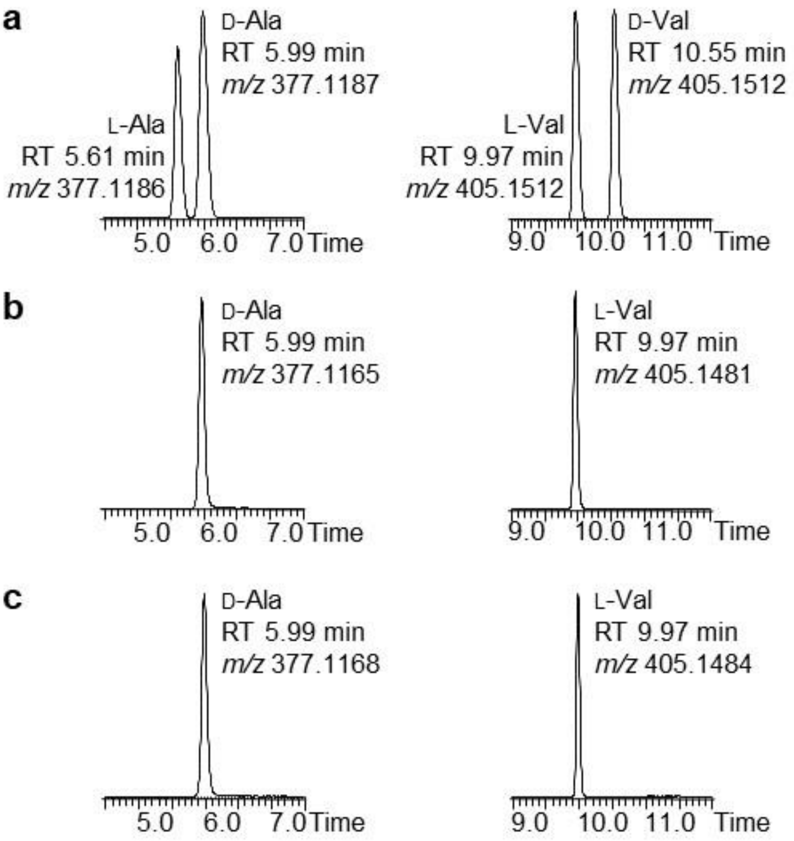

Figure 5. UPLC-ESI$^-$-TOF-MS-chromatograms of acidic hydrolysis after chiral amino acid derivatization of (**a**) amino acid references L- and D-alanine and L- and D-valine, (**b**) cereulide (**1**), and (**c**) isocereulide A (**2**).

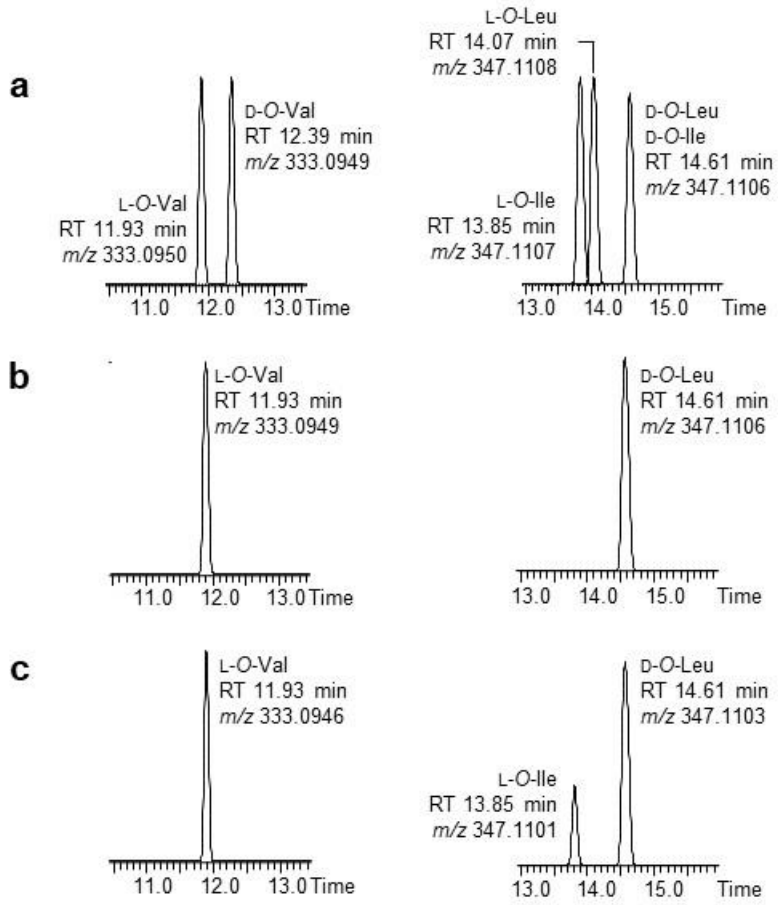

Figure 6. UPLC-ESI^{-}-TOF-MS-chromatograms of acidic hydrolysis after chiral α-hydroxy acid derivatization of (**a**) α-hydroxy acid references L- and D-*O*-valine, and L- and D-*O*-leucine with L- and D-*O*-isoleucine, (**b**) cereulide (**2**), (**c**) isocereulide A (**1**).

A. NMR data of cereulide (**1**) and 1D- and 2D-NMR spectra of cereulide (**1**), and isocereulide A (**2**)

NMR-data of cereulide:

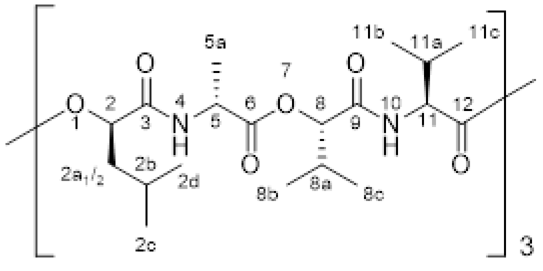

Cereulide (1):

^{1}H-NMR [500MHz. pyridine-d_5, 298K]: ⊚ 0.90 [d, 9H, *J*=6.18 Hz, H_3-C(2c)], 0.97 [d, 9H, *J*=6.37 Hz, H_3-C(2d)], 1.07-1.14 [3 d, 27H, *J*=6.80, 6.86, 6.62 Hz, H_3-C(8c, 11c, 11b)], 1.17 [d, 9H, *J*=6.74 Hz, H_3-C(8b)], 1.68 [d, 9H, *J*=7.29 Hz, H_3-C(5a)], 1.88-2.03 [m, 6H, H-C($2a_1$, 2b)], 2.06-2.16 [m, 3H, H-C($2a_2$)], 2.38-2.50 [m, 3H, H-C(11a)], 2.50-2.61 [m, 3H, H-C(8a)], 4.69-4.75 [t, 3H, *J*=7.03 Hz, H-C(11)], 4.75-4.82 [quint, 3H, *J*=6.82 Hz, H-C(5)], 5.43 [d, 3H, *J*=4.51 Hz, H-C(8)], 5.64 [dd, 3H, *J*=3.04, 9.64 Hz, H-C(2)], 8.79 [d, 3H, *J*=7.22 Hz, H-C(10)], 9.15 [d, 3H, *J*=6.39 Hz, H-C(4)]. ^{13}C-NMR [125 MHz, pyridine-d_5, 298K]: ⊚ 16.9 [C(5a)], 17.2 [C(8b)], 18.7 [C(11b)], 18.8 [C(11c)], 19.2 [C(8c)], 21.1 [C(2c)], 23.1 [C(2d)], 24.5 [C(2b)], 30.1 [C(11a)], 30.8 [C(8a)], 41.0 [C(2a)], 49.4 [C(5)], 58.9 [C(11)], 73.0 [C(2)], 78.6 [C(8)], 170.3 [C(9)], 171.0 [C(3)], 171.5 [C(12)], 172.2 [C(6)].

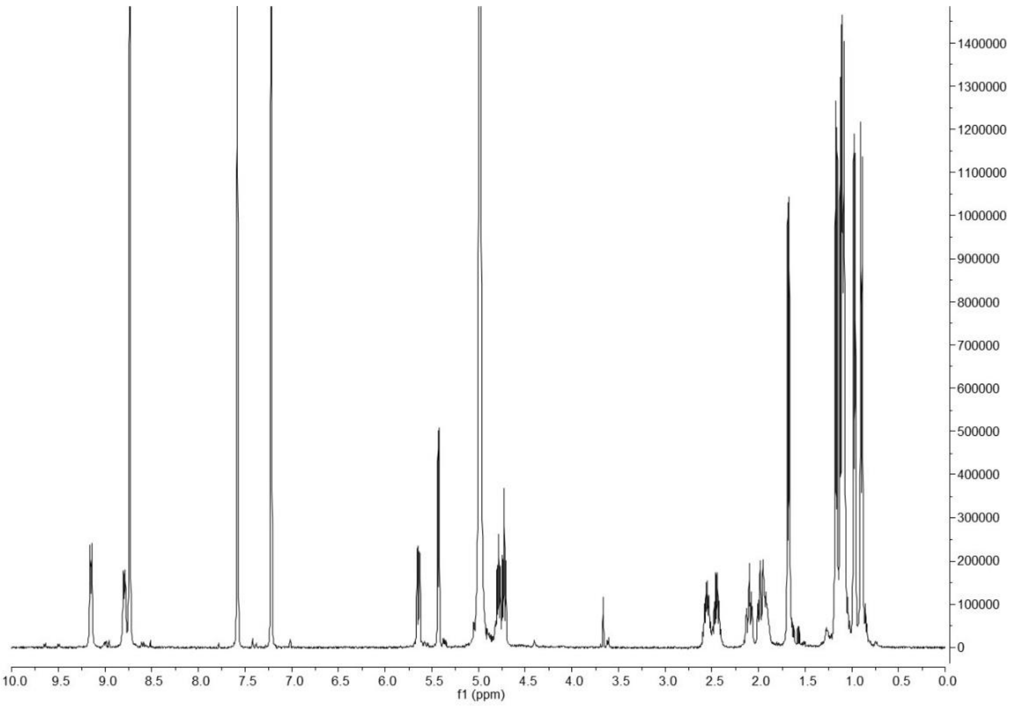

Figure 7. ^{1}H-NMR spectrum of **1** (500 MHz, 298 K, Pyridine-d_5).

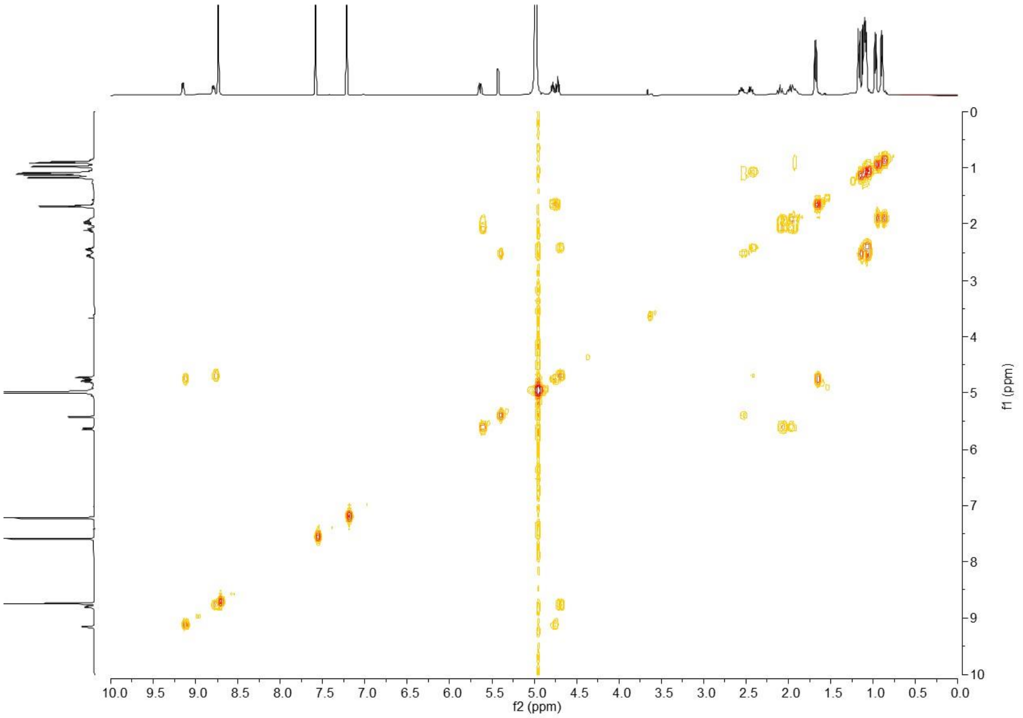

Figure 8. ^{1}H,^{1}H-COSY-NMR spectrum of **1** (500 MHz, 298 K, Pyridine-d_5).

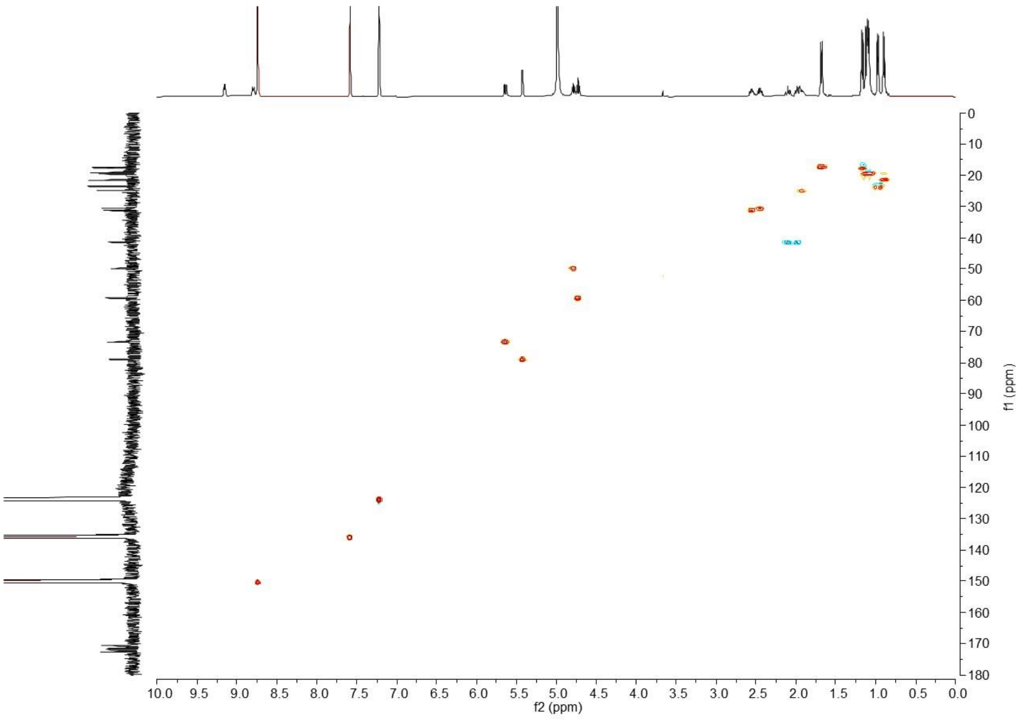

Figure 9. ^{1}H,^{13}C-HSQC-NMR spectrum of **1** (500 MHz, 125 MHz, 298 K, Pyridine-d_5).

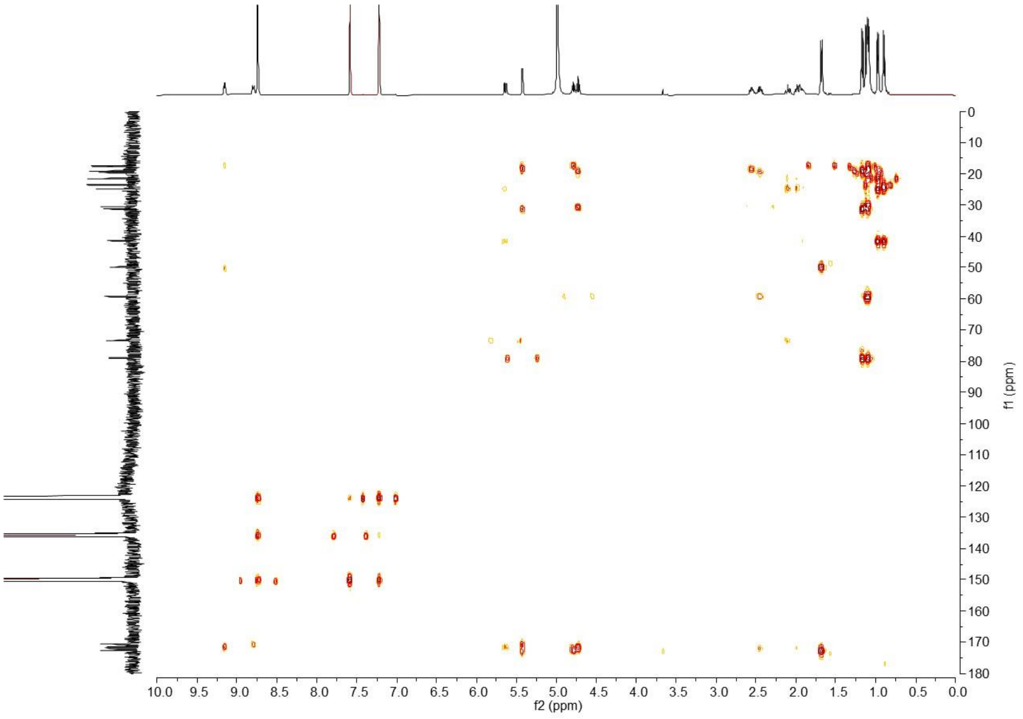

Figure 10. ^{1}H,^{13}C-HMBC-NMR spectrum of **1** (500 MHz, 125 MHz, 298 K, Pyridine-d_5).

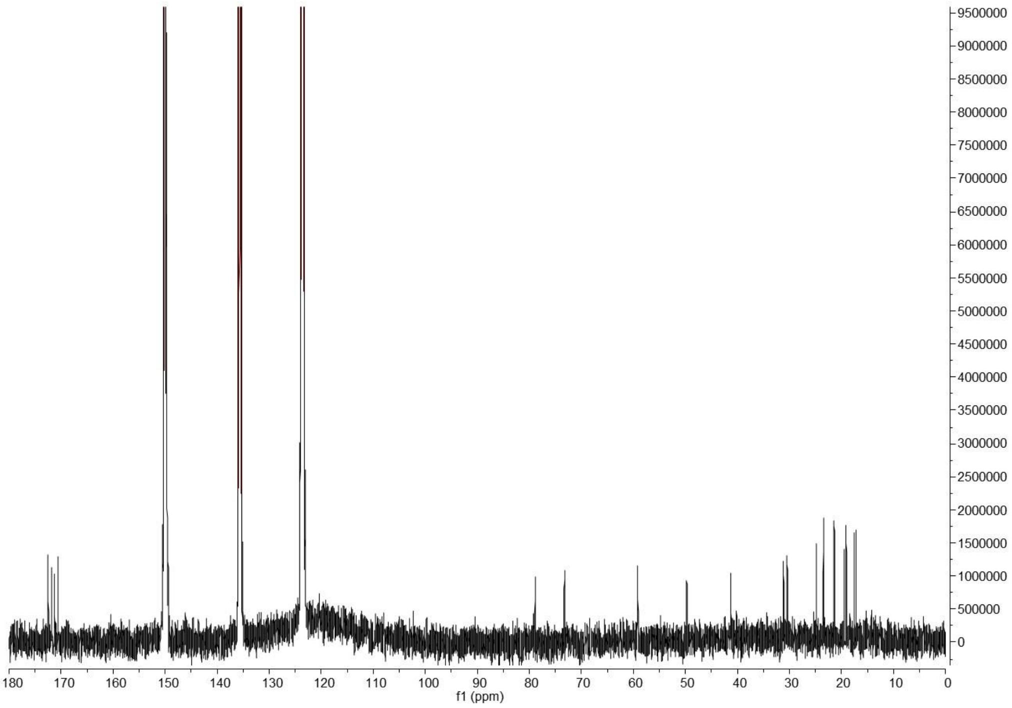

Figure 11. ^{13}C-NMR spectrum of **1** (125 MHz, 298 K, Pyridine-d_5).

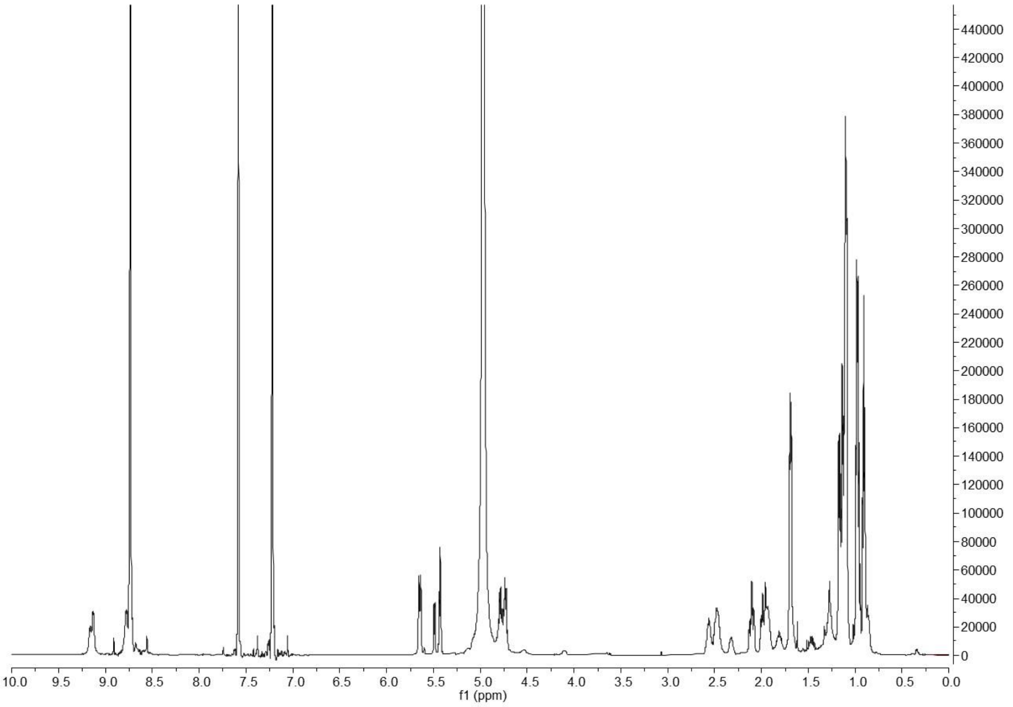

Figure 12. ^{1}H-NMR spectrum of **2** (500 MHz, 298 K, Pyridine-d_5).

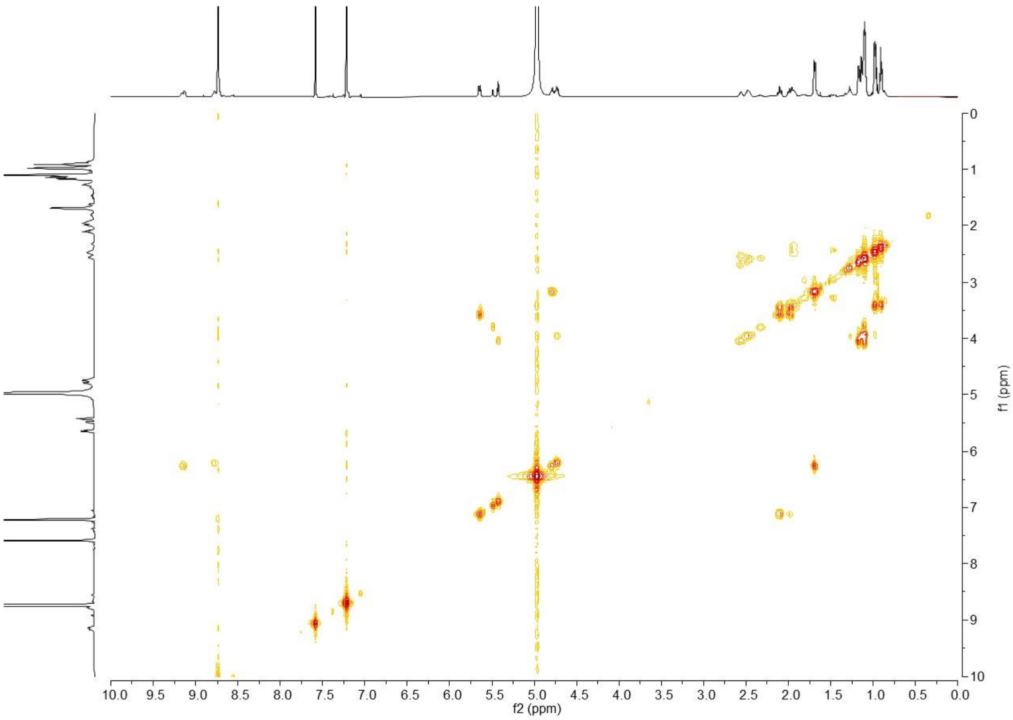

Figure 13. A: $^{1}H,^{1}H$-COSY-NMR spectrum of **2** (500 MHz, 298 K, Pyridine-d_5).

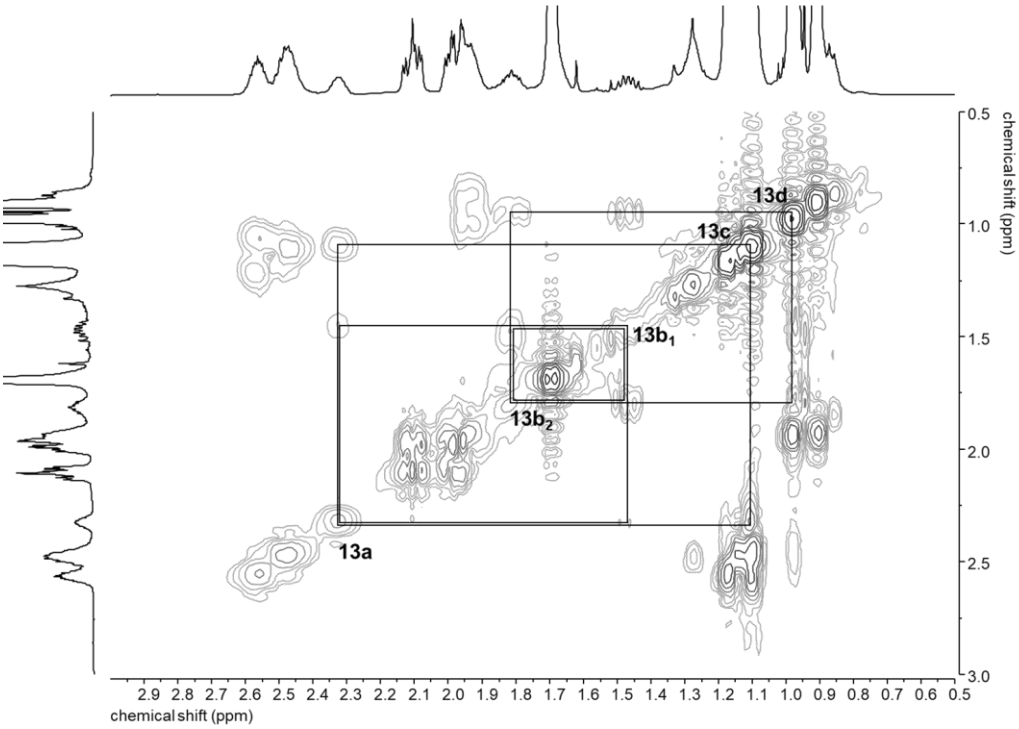

Figure 13. B: Zoom $^{1}H,^{1}H$-COSY-NMR spectrum of **2** (500 MHz, 298 K, Pyridine-d_5).

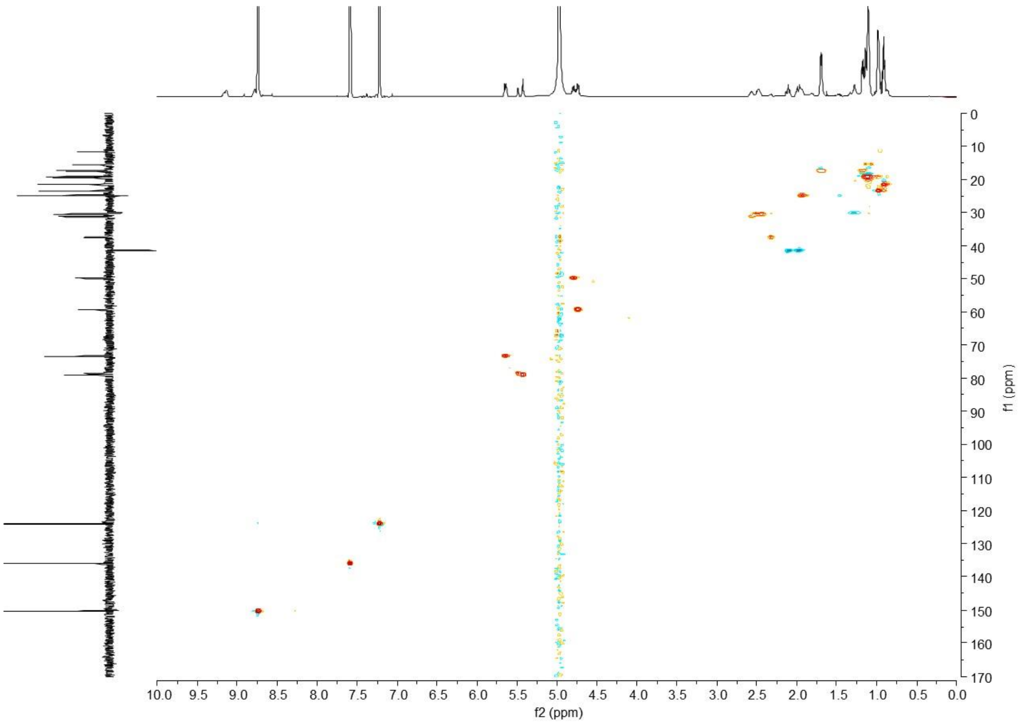

Figure 14. ^{1}H,^{13}C-HSQC-NMR spectrum of **2** (500 MHz, 125 MHz 298 K, Pyridine-d5).

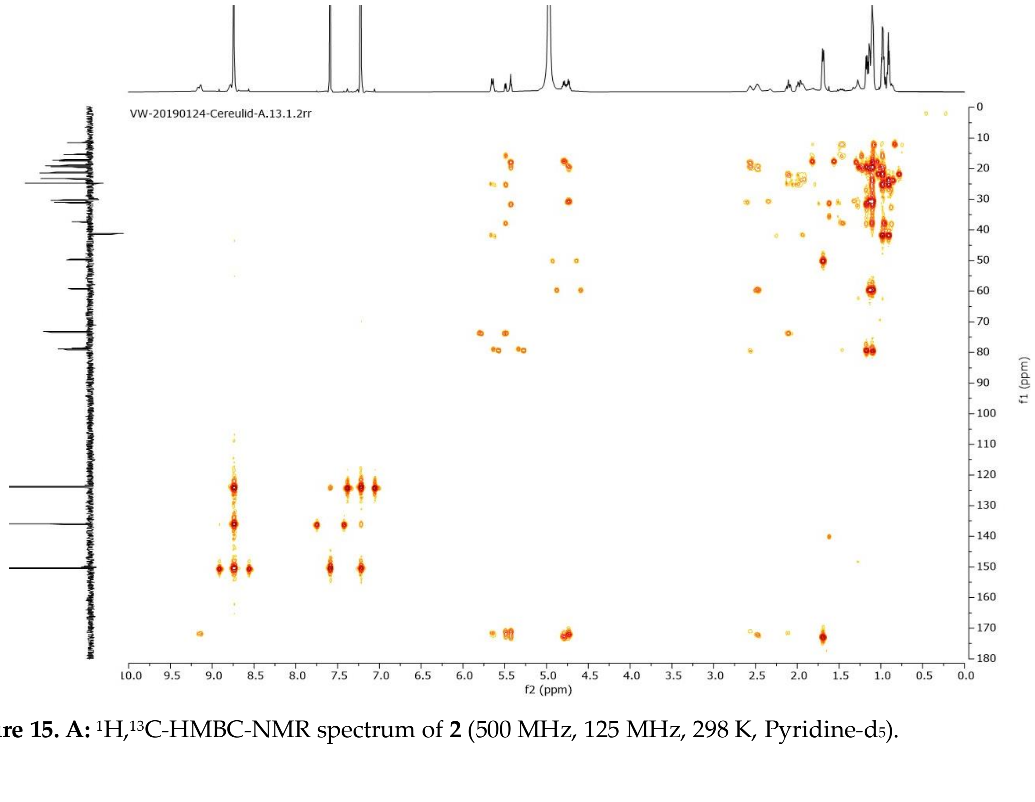

Figure 15. A: ^{1}H,^{13}C-HMBC-NMR spectrum of **2** (500 MHz, 125 MHz, 298 K, Pyridine-d5).

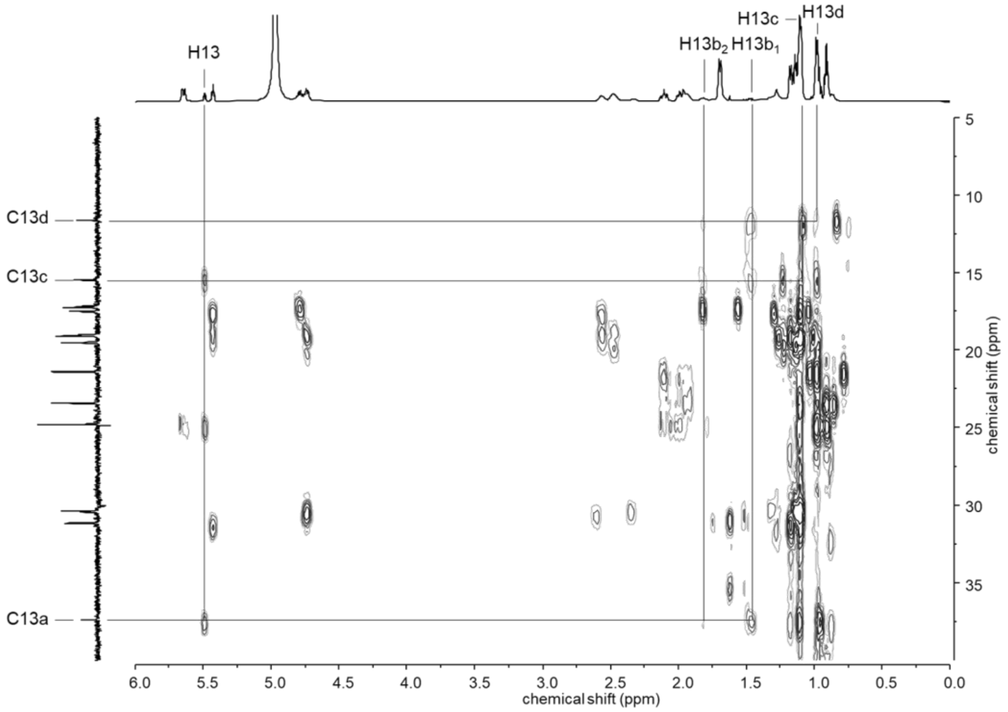

Figure 15. B: Zoom ^{1}H,^{13}C-HMBC-NMR spectrum of **2** (500 MHz, 125 MHz, 298 K, Pyridine-d_5).

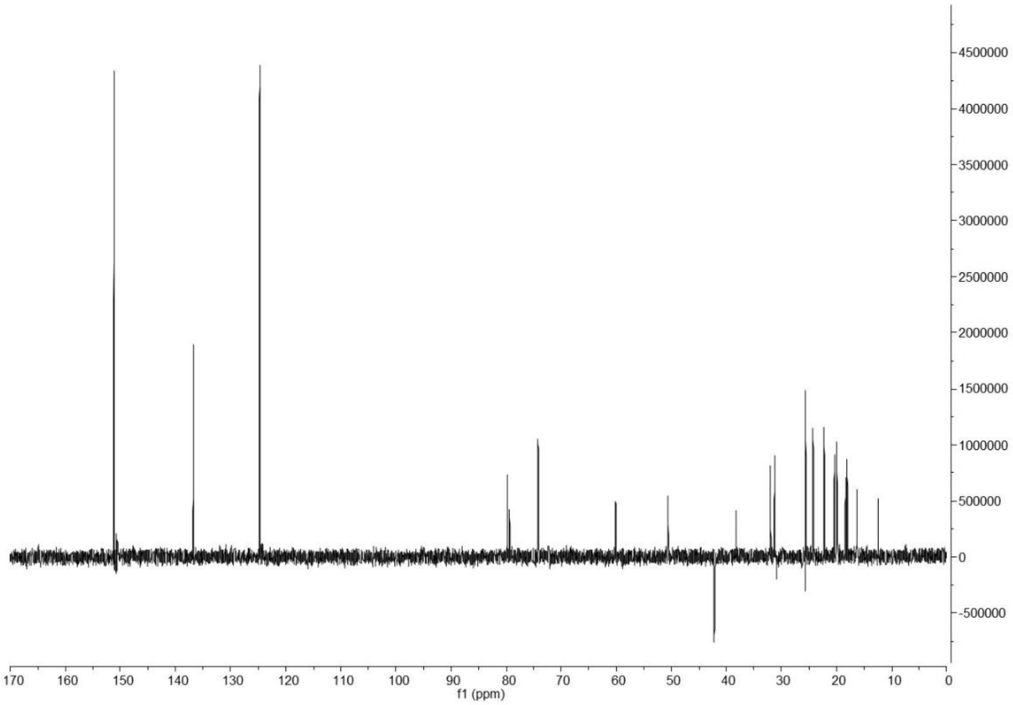

Figure 16. DEPT135-NMR spectrum of **2** (125 MHz, 298 K, Pyridine-d_5).

MS^n-Data of cereulide (**1**) and isocereulide A (**2**)

Cereulide (1):

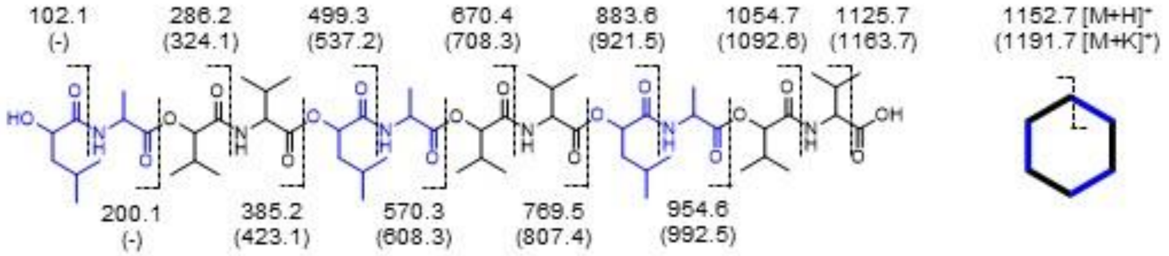

MS:

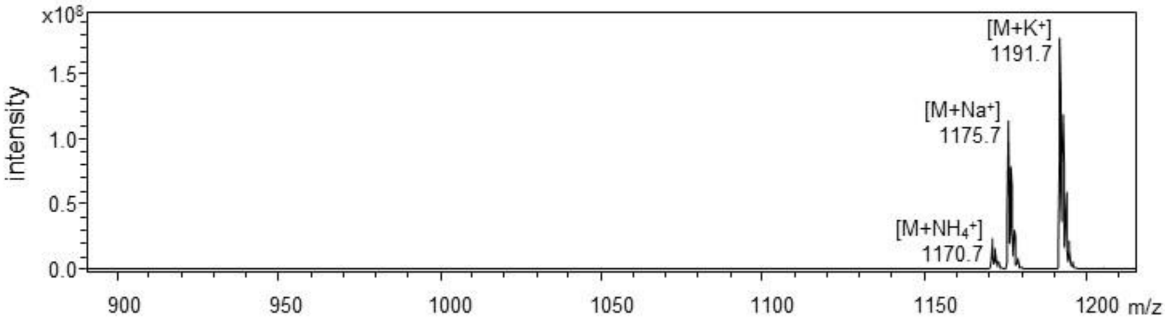

MS^2 (1191.7 → x)

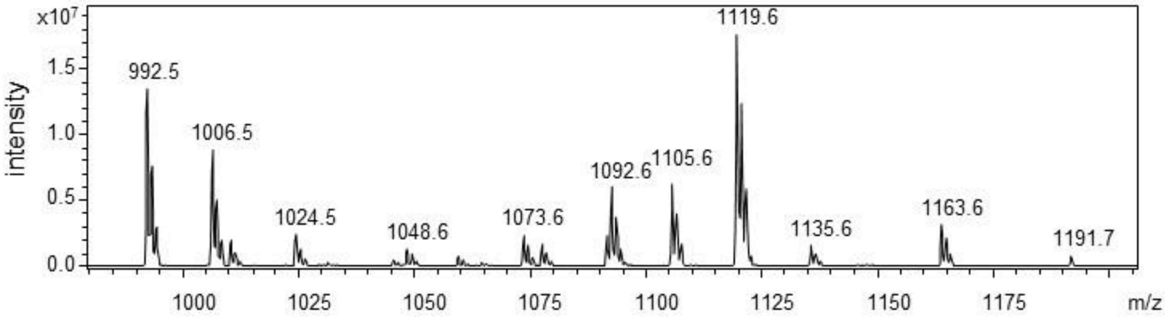

MS^3 (992.5 → x).

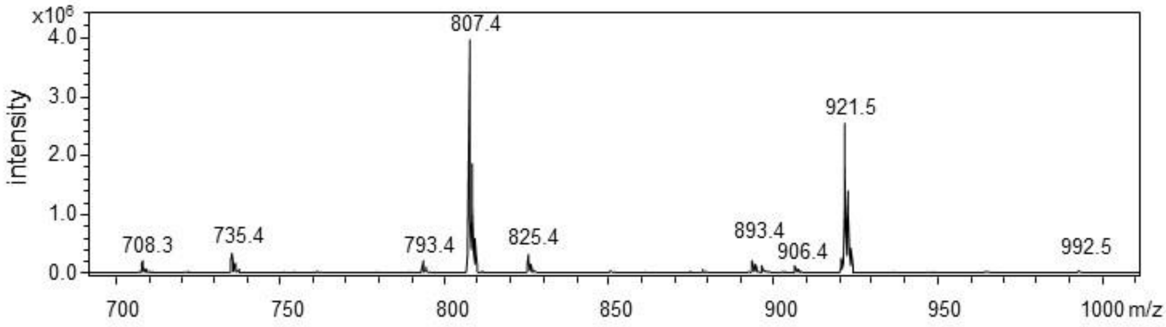

MS^4 (807.4 → x)

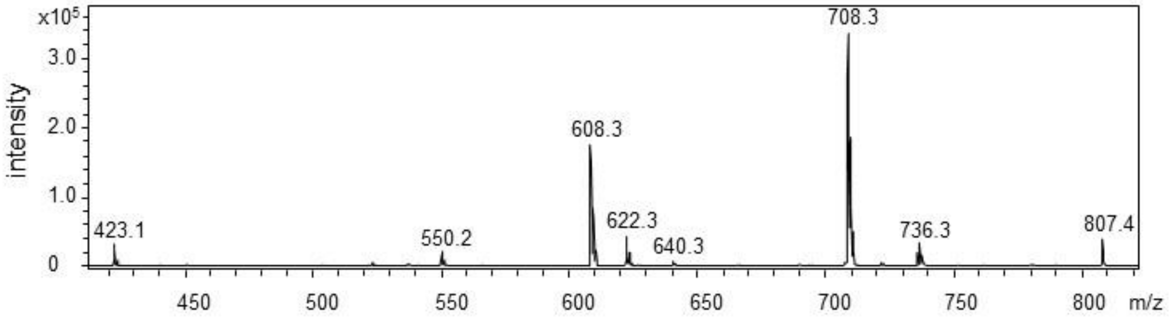

MS^5 (608.3 → x)

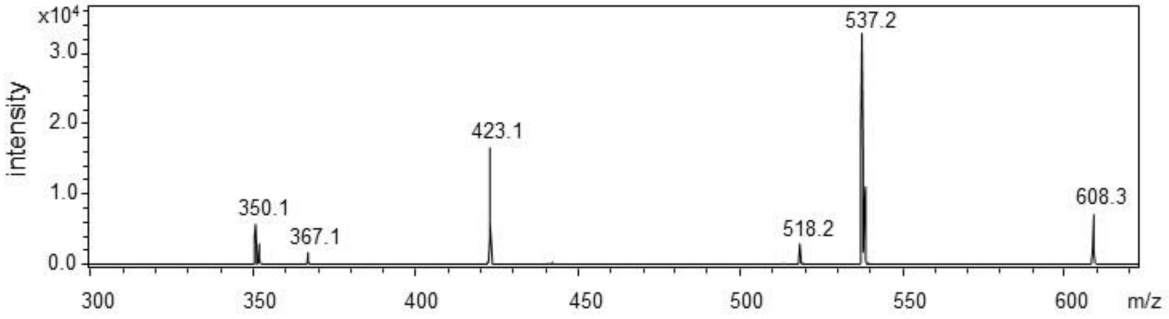

MS^6 (423.1 → x)

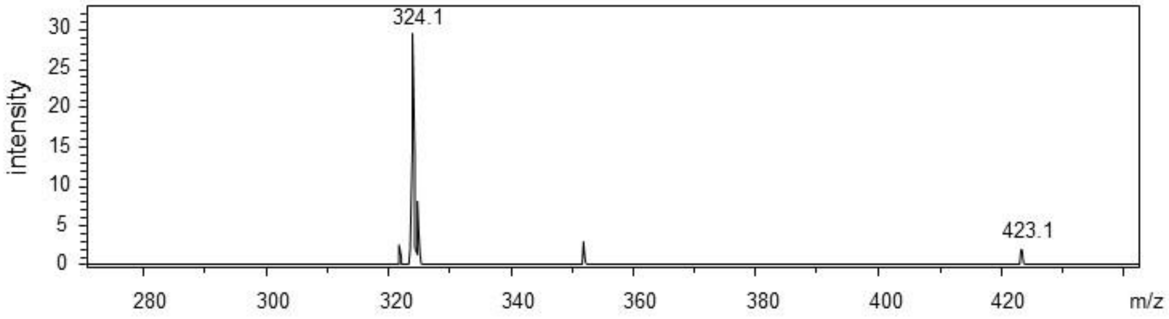

Isocereulide A (2):

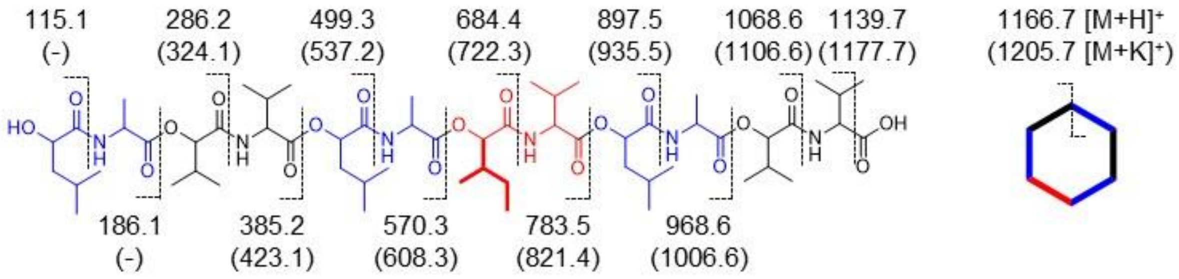

MS:

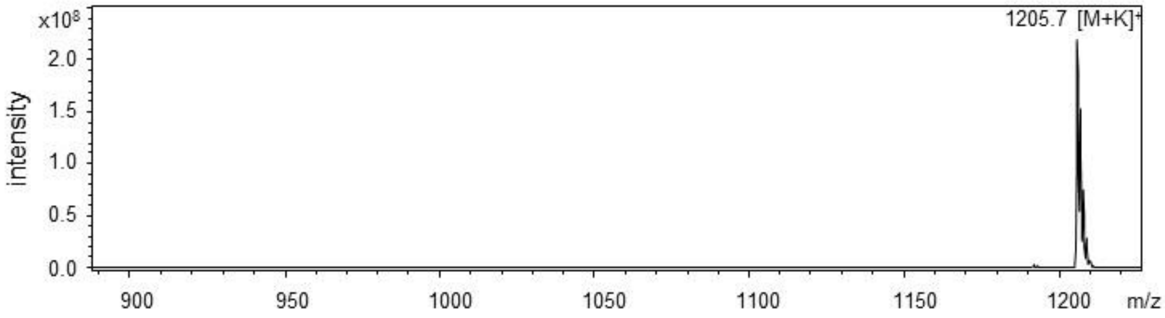

MS^2 (1205.7→ x)

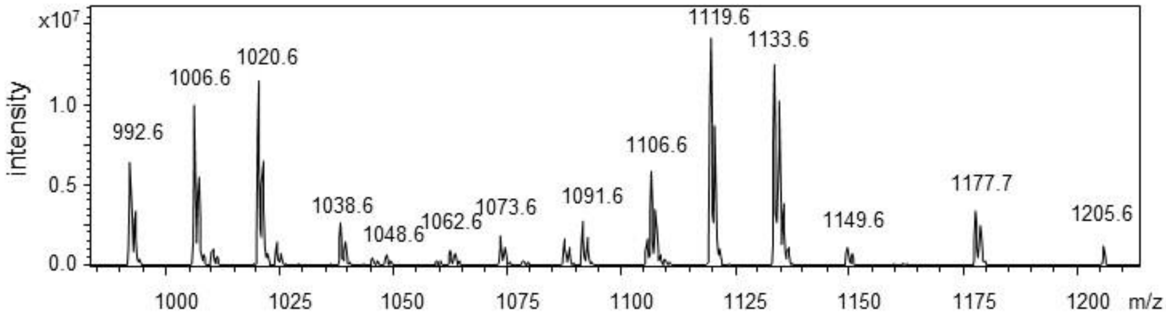

MS^3 (1006.5→ x)

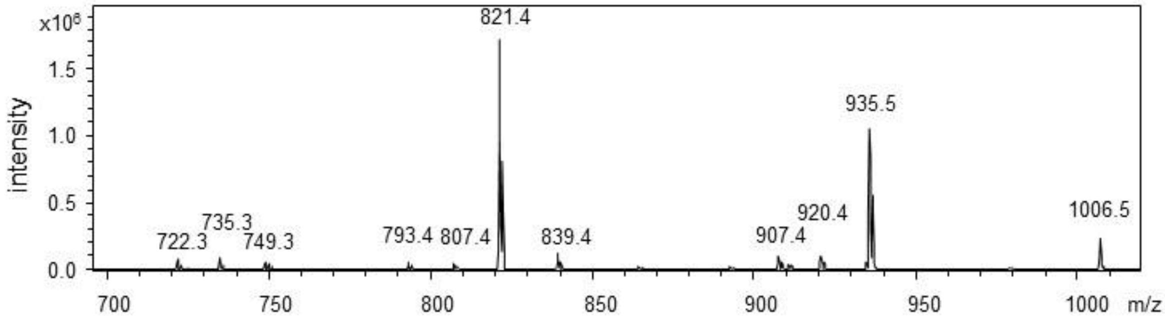

MS^4 (821.4 → x)

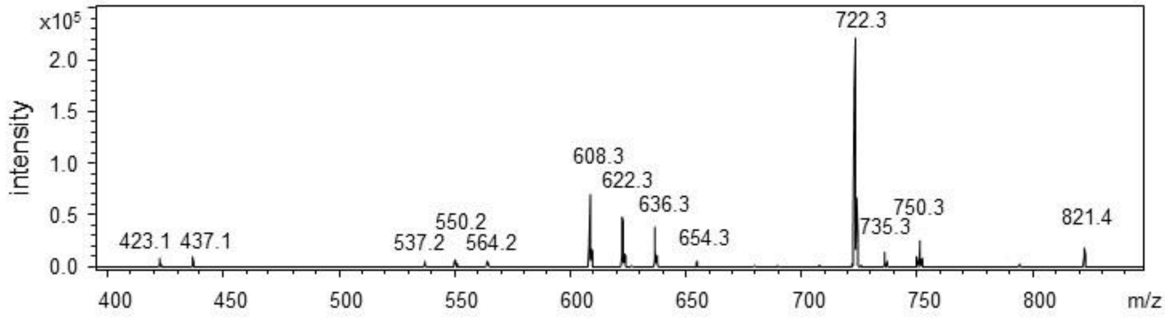

MS[5] (608.3 → x).

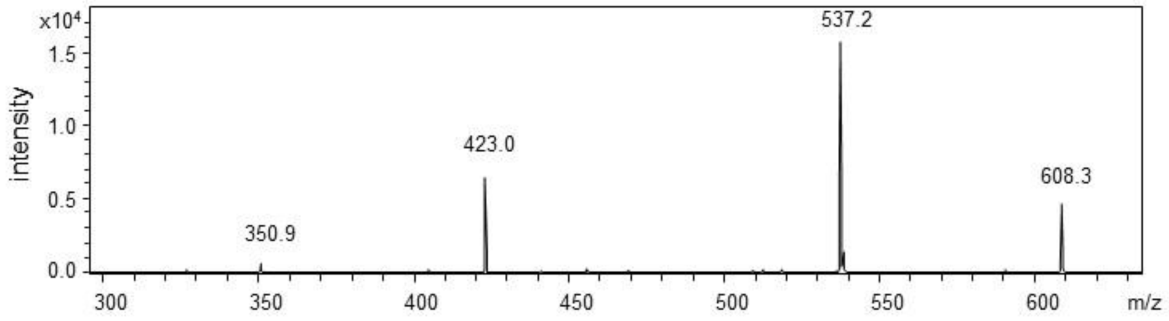

MS[6] (423.1 → x)

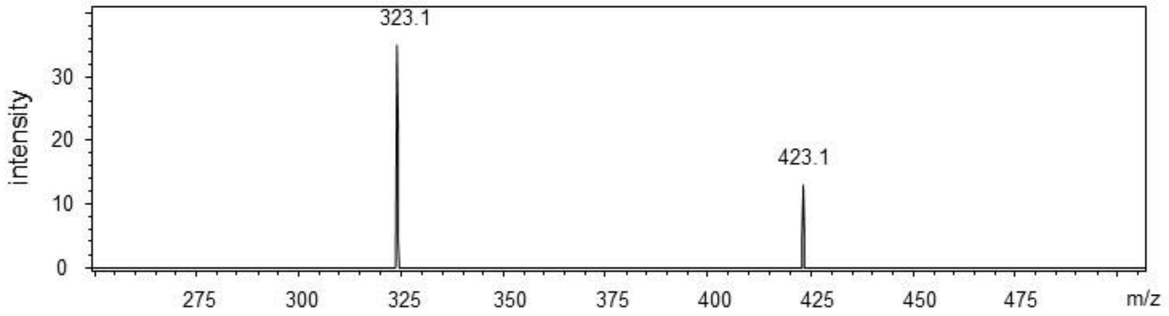

References

1. Marxen, S.; Stark, T.D.; Frenzel, E.; Ruetschle, A.; Luecking, G.; Puerstinger, G.; Pohl, E.E.; Scherer, S.; Ehling-Schulz, M.; Hofmann, T. Chemodiversity of cereulide, the emetic toxin of *Bacillus cereus*. *Anal. Bioanal. Chem.* **2015**, *407*, 2439–2453, doi:10.1007/s00216-015-8511-y.
2. Brückner, H.; Jaek, P.; Langer, M.; Godel, H. Liquid chromatographic determination of D-amino acids in cheese and cow milk. Implication of starter cultures, amino acid racemases, and rumen microorganisms on formation, and nutritional considerations. *Amino Acids* **1992**, *2*, 271–284.
3. Chan, W.C.; White, P.D.; Editors. *Fmoc Solid Phase Peptide Synthesis*. *A Practical Approach;* Oxford Univ Press, 2000.
